# Human Resources for Treating HIV/AIDS: Are the Preventive Effects of Antiretroviral Treatment a Game Changer?

**DOI:** 10.1371/journal.pone.0163960

**Published:** 2016-10-07

**Authors:** Till Bärnighausen, David E. Bloom, Salal Humair

**Affiliations:** 1 Department of Global Health and Population, Harvard T. H. Chan School of Public Health, Boston, Massachusetts, United States of America; 2 Africa Health Research Institute (AHRI), Mtubatuba, KwaZulu Natal, South Africa; 3 Institute of Public Health, University of Heidelberg, Heidelberg, Germany; 4 Amazon.com, Inc., Seattle, Washington, United States of America; Boston University, UNITED STATES

## Abstract

Shortages of human resources for treating HIV/AIDS (HRHA) are a fundamental barrier to reaching universal antiretroviral treatment (ART) coverage in developing countries. Previous studies suggest that recruiting HRHA to attain universal ART coverage poses an insurmountable challenge as ART significantly increases survival among HIV-infected individuals. While new evidence about ART’s prevention benefits suggests fewer infections may mitigate the challenge, new policies such as treatment-as-prevention (TasP) will exacerbate it. We develop a mathematical model to analytically study the net effects of these countervailing factors. Using South Africa as a case study, we find that contrary to previous results, universal ART coverage is achievable even with current HRHA numbers. However, larger health gains are possible through a surge-capacity policy that aggressively recruits HRHA to reach universal ART coverage quickly. Without such a policy, TasP roll-out can increase health losses by crowding out sicker patients from treatment, unless a surge capacity exclusively for TasP is also created.

## Introduction

During the past decade, major progress has been made in enrolling HIV-infected people in antiretroviral treatment (ART) programs. Sub-Saharan Africa exemplifies this progress, where approximately 9 million people received ART in 2013 [1] compared with 100,000 in 2003 [2]. Although HIV treatment has become one of the largest coordinated programs in the history of global public health, large ART coverage gaps remain. In many countries in sub-Saharan Africa, one in two persons urgently needing life-saving ART under the current ART-eligibility guidelines still does not receive treatment [2].

Attaining universal antiretroviral treatment (ART) coverage for HIV-infected ART-eligible people is a central goal of global HIV efforts. The last decade’s global HIV response has substantially addressed shortages of drugs, equipment, and facilities in many developing countries [3, 4]. Still unaddressed, however, is the lack of human resources to treat HIV/AIDS (HRHA) [5–7], arguably the most significant remaining constraint [8–11]. This constraint is not easily relaxed because of several well-known issues, including low capacity of nursing and medical schools in developing countries [10]; high emigration of trained health workers [12, 13]; limited incentives for health worker retention (e.g., salaries and working conditions) in many national health systems [8, 14–20]; and internal and external fiscal constraints on many countries’ development budgets [21].

**Fig 1:**
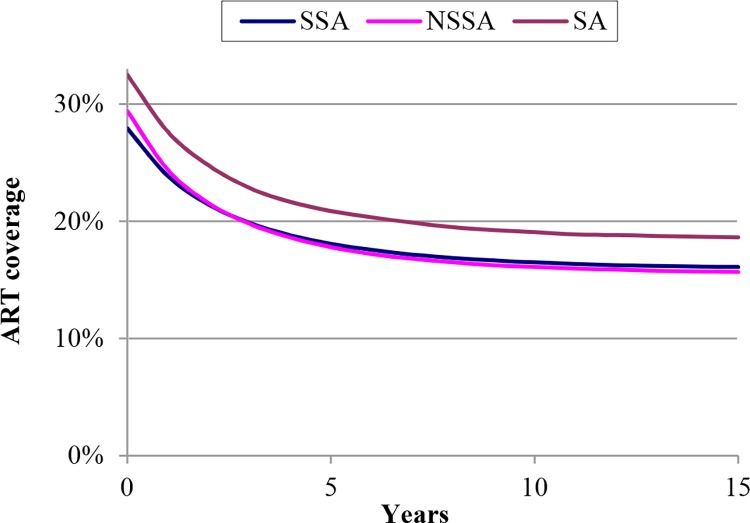
Previous results from (5) showing ART coverage would decline if human resources for delivering ART remained constant. Results are shown for sub-Saharan Africa (SSA), non–sub-Saharan Africa (NSSA), and South Africa (SA). Year 0 is 2007.

However, even if some of these constraints could be mitigated, previous studies have indicated another, more fundamental, problem in overcoming the HRHA barrier [5]. ART is a lifelong treatment that leads to significantly improved survival of HIV-infected people. Thus, higher ART coverage (proportion of ART-eligible patients who receive treatment) leads to more people needing ART in the future. The considerably increased survival produces two dynamics: one, if HRHA numbers were to stay constant, ART coverage would decline over time [5], and two, if HRHA numbers were to increase, every additional fixed increment of HRHA (e.g., 1,000 additional HRHA) would produce a smaller increase in ART coverage than the previous increment [22]. Fig 1 and Fig A in S1 File) highlight the significant effects of these dynamics.

Thus the conclusion from past studies is that in many developing countries with large numbers of HIV-infected people, universal ART coverage cannot be achieved with realistic increases in the numbers of HRHA. This sobering prospect led to phrases such as “victim of its own success” [22, 23], “treatment mortgage” [24, 25], and “ballooning entitlement” [26, 27], referring to the unintended consequences of success in expanding the supply of HRHA—more HRHA available today implies larger number of HRHA needed for further percentage increases in ART coverage.

This conclusion was based on evidence about the large mortality reduction effects of ART [28–30]. This evidence did not, however, account for the effect of ART on HIV transmission, as no clear evidence for such an effect was available then. The results from the HIV Prevention Trials Networks (HPTN) 052 study show the large potential magnitude of this effect—96% reduction in new HIV infections among HIV sero-discordant couples under the conditions of a randomized controlled trial [31]. It is possible that the population-level effectiveness of ART in preventing new infections may be lower than that observed in the HPTN 052 study, because the trial was conducted within a highly motivated ART-adherent group, whereas in general populations about a quarter of patients do not adhere adequately to ART [32, 33]. Nonetheless, emerging evidence indicates ART coverage is also highly effective in reducing HIV incidence in general populations in sub-Saharan Africa [34, 35]. What is not yet clear is how the dynamic interplay between HRHA availability and the mortality and HIV transmission reduction due to ART will affect ART coverage and hence the incidence and prevalence of HIV.

We assess the implications of the HIV-prevention effects of ART in a dynamic setting on (a) the ART coverage that existing HRHA can maintain; (b) the HRHA needed to attain and maintain universal ART coverage; and (c) the population-level health consequences of implementing treatment-as-prevention (TasP) policies, i.e., offering immediate treatment to all HIV-infected individuals to maximize the preventive benefits of ART in addition to the direct health benefits for ART patients. By ART coverage, we mean the proportion of HIV-infected people with CD4 cell counts less than 350 cells/μl who receive treatment. The 350 cells/μl threshold follows the WHO 2010 guidelines for initiating treatment, which are currently or were until recently implemented in most countries in sub-Saharan Africa [36]. By TasP, we mean treating HIV-infected individuals with CD4 cell count above 350 cells/μl, and by ART we mean treating people with CD4 cell count under 350 cells/μl. Alternative definitions of TasP are possible [36], but this definition leads to the most conceptual clarity for the purposes of our analysis. It is also consistent with broad policy usage, such as in the WHO endorsement of TasP as a future strategy for worldwide HIV treatment [37]. Finally, in the interest of comparability with previous studies, we define universal coverage as 100% of those in need of treatment receiving ART. However, we do explore several scenarios in which only 80% or less of those needing treatment receive the benefit of ART.

Using South Africa as a case study, our analysis has generated three key findings. First, contrary to previous results, our analysis suggests that even if HRHA numbers stay constant, ART coverage will slowly increase rather than decline, as the prevention effects of ART will outweigh the mortality reduction effects of ART at the population level. While this result is encouraging, the magnitude of increase in ART coverage due to ART prevention is slight. Consequently, expanding HRHA availability remains an imperative for quickly reaching universal ART coverage. While this challenge seems daunting, the second key finding from our analysis is that such expansion does not need to be maintained in perpetuity. In fact, creating a *surge capacity* in the HRHA workforce would allow for reaching universal ART coverage relatively soon, followed by transitioning to significantly lower HRHA levels over time—all of which the HIV prevention effects of ART make possible. Creating and transitioning this surge capacity is possible within a single decade. Our third and final key finding concerns an important caution in the use of limited human resources. *If* significantly increasing the number of HRHA is not possible, implementing TasP has the potential unintended consequence of *crowding out* sicker HIV patients from ART access, leading to many avoidable deaths and new infections over the next decade.

### Motivation and analysis framework

In addition to new evidence about the HIV-prevention effects of ART, multiple reasons motivate a fresh assessment of HRHA needs. Spurred by concerns about sustainability, global HIV programs are undergoing significant transitions and many donor-funded ART programs are being integrated into country-level health systems [38–41]. These transitions raise concerns for retention of existing HRHA and recruitment of additional HRHA [25, 38, 41], but how much such concerns have been factored into transition plans is unclear [42]. Our results suggest policy guidance for such transitions. Importantly, evidence about the preventative benefits of ART [31, 34] has generated significant momentum for using TasP [43, 44], and the WHO recently announced that it will endorse TasP as a universal strategy for the provision of ART worldwide [37]. While TasP policies clearly will create even larger need for HRHA, the impact of implementing TasP with currently available HRHA on health outcomes has not been examined. We investigate these questions analytically for the first time.

Two factors complicate this investigation: opposing effects of the same interventions (such as ART, or TasP) on the HRHA needed and the dynamic interaction of multiple HIV interventions (such as ART and TasP together or in addition to other interventions such as male circumcision). For instance, for ART, the notions of “victim of its own success” or “treatment mortgage” lead us to expect that universal ART coverage will become more difficult to achieve as we increase HRHA numbers; however, reduction of new HIV infections due to ART may partially or completely offset this difficulty. When ART and TasP are implemented together, another complication occurs as increased ART coverage affects the number of people eligible for TasP (through fewer infections), and increased TasP coverage reduces the number of people needing ART over time (as fewer people progress to CD4 count less than 350 cells/μl). But these cross-linked needs are spread out over time, which complicates guessing *a priori* the net effect of different levels of ART and TasP coverage.

Fig B in S1 File presents empirical evidence that illustrates the complicated dependencies between ART and TasP. The figure, based on a population-based survey of a rural population in South Africa [45], shows how the provision of ART significantly changes the distribution of HIV-infected people across different CD4 categories, thus changing the level of resources needed for ART and TasP in the future. Although this figure provides only a snapshot of one intervention from South Africa where the ART-eligibility threshold was CD4 cell count <200μl, it shows an approximate doubling of the proportion of HIV-infected population with CD4 cell count <200 μl because of ART provision. This suggests significant changes can occur in the distribution of HIV-infected people when ART and TasP are implemented together, and underlines the difficulty of positing *a priori* how simultaneous rollout of these interventions may affect their respective resource requirements.

Several models have been independently developed to understand the evolution of the HIV epidemic and to project the impact of different HIV-related interventions. These models, spanning both micro-simulations that focus on an individual’s life trajectory and compartmental models that focus on cohorts of people, incorporate rich details and varying representations of complex relationships in the HIV epidemic, such as different age-mixing patterns in sexual partnerships, age profiles of sexual behavior, or concurrent sexual partnerships [46–54]. These models have greatly helped in exploring the population-level impact of expanding ART programs in many settings. In addition, they have different strengths depending on the questions they were designed to answer, different levels of data requirements, and usually require statistical calibration by fitting a curve to the observed history of the disease.

To address specific questions regarding the effect of alternative health workforce resource allocations for HIV treatment, we needed to develop an appropriate model of the HIV epidemic. Standard models of the HIV epidemic assume that the rates of change of population sizes at different disease stages are proportional to the overall population sizes. This assumption is not tenable under alternative distributions of a fixed number of health workers across patients in different disease stages. Instead, we needed a model of the epidemic in which the rate of change of population size at each disease stage is proportional not just to the population size, but to the minimum of the available resources and the population size. To this end, we built a new causal theory-based model of the HIV epidemic (further discussion in the S1 File). One virtue of our model is its closed-form representation. Instead of being based on a curve that is fit to historical data on the history of the HIV epidemic, the model is based on a parsimonious set of assumptions that reflect well-established biological and behavioral mechanisms of HIV transmission. This structure allows us to translate different distributions of absolute numbers of HRHA into epidemic trajectories. A second virtue is that the model exhibits a high degree of transparency: given the initial conditions, replicating our results by repeatedly applying the supplied mathematical equations is easy (more details are provided in the S1 File).

For purposes of the analysis, we need an appropriate definition of HRHA. Provision of ART to a typical patient involves a team of physicians, nurses, ART counselors, and pharmacy staff. The range of ART-related tasks performed by these cadres is quite broad, including pre-ART counseling, establishing medical eligibility for ART, initiating ART, monitoring patients for side effects, responding to new signs and symptoms, managing drug substitution, etc. [55]. A typical new patient at an HIV clinic is counseled about HIV and ART and evaluated for disease status and ART eligibility. Patients who are initiated on ART are requested to visit the clinic for reassessment two weeks after initiation and subsequently at monthly intervals. For patients on ART, repeat CD4 cell count tests and viral load tests, if available at the clinic, are performed either quarterly or every six months [56]. Except for pre-ART counseling and some aspects of clinical monitoring (e.g., taking weight, checking adherence), all these tasks require nurse or physician involvement [55]. Consequently, many ART programs in South Africa are now nurse-led [57].

While variation exists in the composition of HRHA teams and the ART-related tasks performed at each facility, aggregate data tend to smooth out the variability. Reasonable aggregate data about the composition of HRHA teams are available for South Africa from studies of staffing patterns and practices in ART programs [56, 58, 59]. For instance, Hirschhorn et al. [58] find that 1–2 doctors, 1–3 pharmacy staff, and 2–7 nurses are needed to provide ART to 1,000 patients; Filler at al. report from a study of 45 clinics supported by the Presidential Emergency Plan for AIDS Relief (PEPFAR) that the median number of physicians providing ART to 1,000 patients was 1.29 [56]; and Hagopian et. al., at the time of their study in 2008, find on average 1 physician provided ART to 2,155 patients in Mozambique [60].

We therefore define one HRHA unit as a team that can provide ART to 1,000 patients per year. The results of the model are thus insensitive to the precise make-up of the team. By multiplying the number of HRHA teams needed by appropriate scale factors, we can apply the model results to physicians, nurses, or any other health worker cadre whose involvement is needed in the provision of ART. The actual HRHA team composition for a country will depend on the organization and extent of task shifting in its ART program [61]. For example, if 100 HRHA are needed for universal ART coverage, then a country with largely physician-led programs for which 1.29 physicians are required per 1,000 patients would require 129 physicians for universal coverage. But in a country with nurse-led programs where only 0.2 physicians are required per 1,000 patients, only 20 physicians would be required for universal coverage. Task shifting can then be represented as changing ratios of health worker cadres required to deliver ART to 1,000 patients. The flexibility to examine HRHA needs independent of task shifting offers an advantage in studying countries where experiments with the shifting of tasks from physicians to nurses and deliberations about further shifting of tasks to community health workers are ongoing.

## Materials and Methods

**Fig 2:**
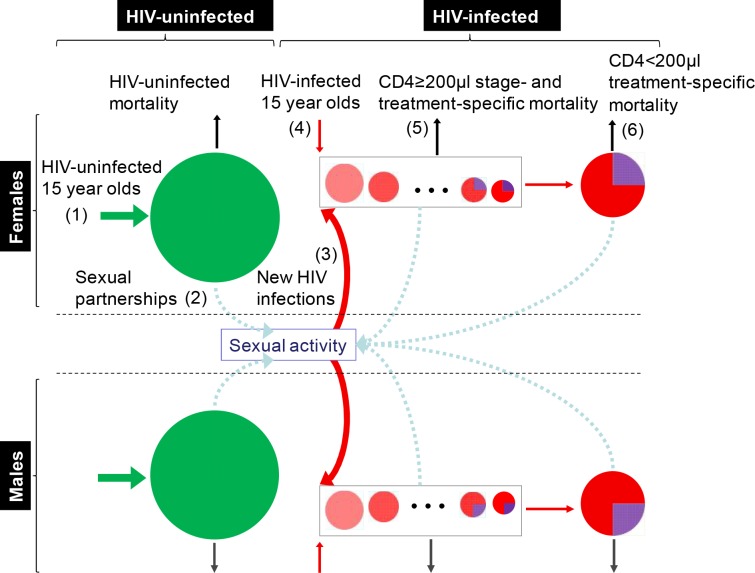
The HIV infections model. New 15-year-old HIV-uninfected individuals flow into the HIV-uninfected pools (1). HIV-uninfected individuals participate in sexual activity with HIV-uninfected and HIV-infected individuals (2), giving rise to new HIV infections (3), which together with new HIV-infected 15-year-olds (4) add to the HIV-infected pool. HIV-infected people progress through different stages of HIV infection (5) until reaching CD4 cell count < 200μl (6) (purple color indicates people receiving ART).

We formulate a discrete-time mathematical model with yearly time increments and two main population classes: men and women aged 15 and older (Fig 2). Each population class is divided into people without HIV infection and HIV-infected people differentiated by the number of years since HIV acquisition. The years since HIV acquisition model the decline in CD4 cell count over time, e.g., 5 years after infection, an untreated person’s CD4 cell count falls below 350/μl. HIV-uninfected men are further subdivided into pools of circumcised and uncircumcised men, while HIV-infected men are further subdivided into pools of those uncircumcised and not receiving ART, uncircumcised and receiving ART, circumcised and not receiving ART, and circumcised and receiving ART. HIV-infected women are subdivided into pools of those receiving ART and those not receiving ART. Pools can differ from each other through their mortality rate, HIV-transmission probability per sex act, and HIV-acquisition probability per sex act. These parameters depend on the treatment (and for men, circumcision) status of individuals.

Each year, new sexually transmitted HIV infections are computed under the following assumptions: each HIV-negative person has a probability distribution for the number of partners over the year, each partner is randomly drawn from the pools of opposite sex partners, each partner engages with him or her in a given number of sex acts, and each sex act with an HIV-infected partner can result in an infection independently of all other sex acts during the year. In addition to sexual infections, exogenous inflows of new 15-year-olds also occur into the pool of HIV-uninfected individuals and the pool of HIV-infected people in the first year of their infection. The precise mathematical formulation of this model and the data sources used for model inputs are provided in the S1 File.

Given the number of new sexually transmitted infections in a year, each pool subdivision is reduced by its respective mortality, all pools of HIV-infected people not receiving ART are moved one year forward (all pools receiving ART are left in their current year), the number of people receiving TasP/ART in each pool is updated based on the resources available for the next year, and new additions are made to the pools of HIV-uninfected and HIV-infected individuals.

In our model, circumcision coverage and condom usage are kept constant at current levels. ART provided to every HIV-infected person with CD4 cell count ≥350/μl is considered TasP, and ART provided to every person with CD4 cell count <350/μl is considered treatment. In one set of scenarios, allocation of HRHA is proportional to the fraction of the population in each pool, i.e., HRHA are allocated so that the fraction of people receiving TasP and ART within a pool equals the target coverage levels. In other scenarios, HRHA are distributed across HIV disease stages differentially: more HRHA are allocated to more advanced disease stages, reflecting ART-initiation patterns in many developing countries.

One of the innovations in our model is that it allows the translation of a resource allocation into an epidemic trajectory. In a past study we have used this model to establish the comparative cost-effectiveness of alternative combinations of several HIV prevention interventions [62]. Here, this model capability allows us to determine the impact of reallocating a constant number of health workers to different patient distributions in the different HIV disease stages. This feature allows us for the first time to establish how health outcomes change when scarce health workers are shifted from treating HIV patients in advanced disease stages to treating those in earlier disease stages, showing the “crowding out” effects discussed in the paper.

The model has been compared with multiple other models, which have different underlying structures, with respect to the impact of different types of HIV interventions: the results from our model conform closely to those from these other models [52, 54]. The mathematical formulation of our model and all the data sources used for model inputs are provided in the S1 File.

## Results

**Fig 3:**
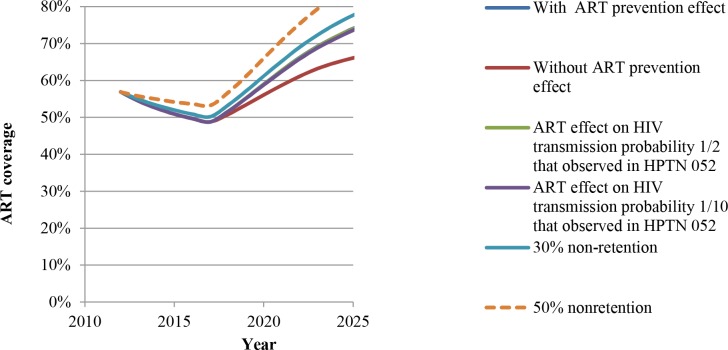
Trajectory of ART coverage in South Africa if human resources for treating HIV/AIDS (HRHA) were to stay constant at the initial level. Coverage shown for the following cases: assuming ART does not have prevention effects; assuming ART prevention effects are as observed in the HPTN 052 trial; ART prevention effects that are 1/2 or 1/10th of that observed in the HPTN 052 trial; and assuming ART non-retention rate is 30% or 50%. The lines for the following three cases are very close and hence all lines are not visible: with ART prevention effect, ART effect on HIV transmission probability 1/2 of that observed in HPTN 052, and ART effect on HIV transmission probability 1/2 of that observed in HPTN 052.

Our first main result (Fig 3) is that if the number of HRHA in South Africa stays constant at the current level, ART coverage (treatment for patients with CD4 cell count <350 μl) will rise rather than decline over time, unlike the result indicated in previous studies [5, 22]. This is because of the negative effect of ART on HIV incidence. This effect is strong enough to compensate for the increased survival of patients on ART and allows reaching universal coverage in approximately 25 years even with current numbers of HRHA (as shown in Fig C in S1 File).

Fig 3 underscores the importance of this finding by also showing the trajectory of ART coverage for a very strong counterfactual comparison: an optimistic scenario that assumes HIV incidence in South Africa is already declining enough to cause increasing ART coverage over time, even without the preventive effects of ART. If HIV incidence is instead roughly constant without the preventive effects of ART, our result would appear even stronger because in that case, ART coverage would decline as shown in Fig 1 from Bärnighausen et al. [5].

The additional ART coverage due to ART’s preventive benefits is approximately 5% higher than the counterfactual in 10 years, about 15% higher in 20 years, and reaches universal coverage within 25 years (Fig C in S1 File). Further, the rise in ART coverage due to the preventive effects of ART is robust to variations in the transmission probability assumed for people on ART who have CD4 cell count >200 μl and also to ART retention and adherence rates (Fig 3). The robustness of our results is further explained in the section on sensitivity and scenario analysis in the S1 File.

**Fig 4:**
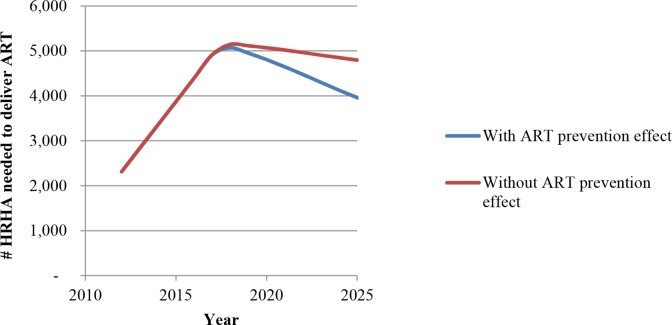
“Surge capacity”: The number of HRHA needed after reaching universal (100%) ART coverage. The Fig shows the drawdown in HRHA that is possible while maintaining universal ART coverage for two cases: if ART has no prevention effects and if ART has prevention effects.

Our second main result is that the number of HRHA needed to maintain universal ART coverage can be significantly reduced *after* universal coverage has been achieved, due to the preventive effects of ART. Fig 4 shows the ART coverage trajectory in South Africa, if the per-year HRHA increases of the past few years continue until universal coverage is attained (Fig D in S1 File shows this trajectory over a longer horizon). In this case, universal coverage is attainable within the next five years, with or without accounting for the preventive effects of ART. In the counterfactual case in which ART has no preventive benefits, HRHA numbers required to maintain universal coverage fall by about 22% in 25 years from the level when universal coverage was attained. However, in the case where ART has preventive benefits, HRHA numbers fall by about 54% in 25 years—within six years of attaining universal coverage they can be drawn down by 20% and within 15 years by about 40%. By 2040, no more than the current numbers of HRHA should be required to maintain universal coverage. The peaking of HRHA needs in Fig 4 and Fig D in S1 File suggest the idea of creating a *surge capacity* for minimizing population health losses due to HIV. This involves scaling up HRHA rapidly to reach universal coverage and then reducing (and redeploying) HRHA progressively to take advantage of the declining need.

**Fig 5:**
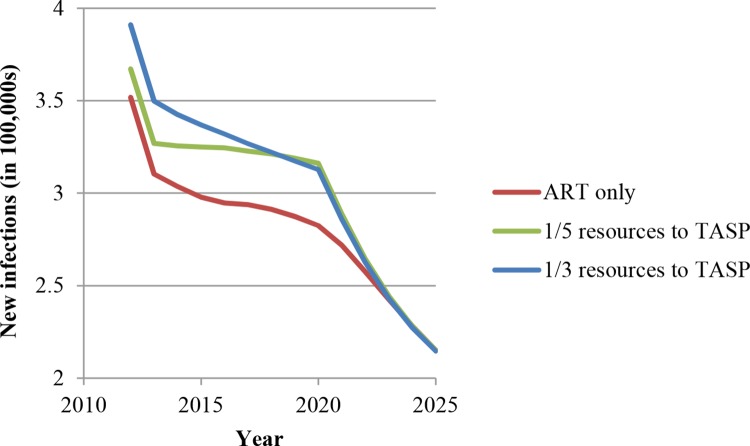
“Crowding out”: Number of new infections per year in South Africa if a fixed number of HRHA are diverted from ART to TasP.

**Fig 6:**
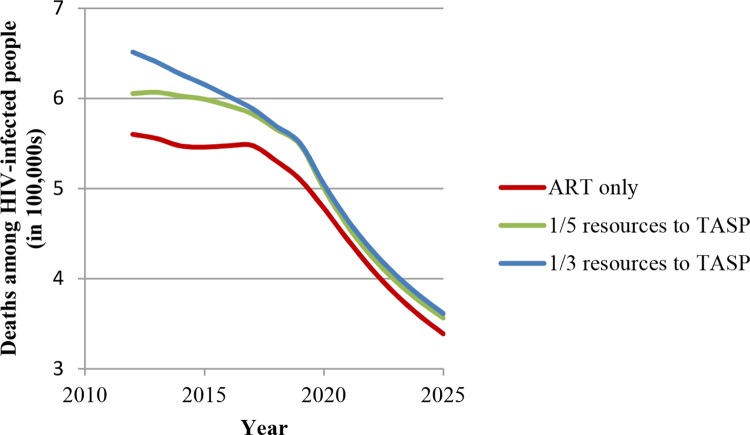
“Crowding out”: Number of HIV-related deaths per year in South Africa if a fixed number of HRHA are diverted from ART to TasP.

Our third main result is that without substantial HRHA investment, TasP can lead to significant losses in terms of population health outcomes in the medium term (10–15 years). Figs 5 and 6 show the increase in the number of new infections per year and in the number of HIV-related deaths per year if TasP is implemented with the current level of HRHA—if one-fifth or one-third of these HRHA previously delivering ART are shifted to initiating ART for patients in early disease stages. We call this phenomenon *crowding out* of sicker patients. Intuitively, the population health losses due to this *crowding out* make sense, because patients in later stages of HIV have higher mortality risk and are more infectious and thus benefit more from ART in terms of survival and HIV transmission reduction than patients in earlier disease stages. Figs 5 and 6 also show that these population health losses, measured in comparison with the scenario in which HRHA are dedicated to ART only, will no longer continue to accrue after 2025. This finding is due to the fact that, eventually, patients who were initiated in later disease stages (and were subsequently *crowded out* from ART) die and also to the fact that these avoidable deaths free up resources for initiating treatment in earlier disease stages among the surviving HIV-infected people without continued crowding-out effects. As more HRHA are directed to earlier disease stages, fewer people progress to advanced disease stages, and over time the population health losses stop accruing. Although crowding out of sicker patients may appear a transient phenomenon, the large number of avoidable deaths it causes indicates its importance. The policy implication is that a surge-capacity approach to implementing TasP would be beneficial: HRHA are scaled up rapidly to deploy TasP, and, as the sicker patients leave due to natural mortality and fewer patients get sick because of the benefits of TasP, the surge capacity for TasP can be reduced within 10 years.

### Additional scenario analyses

The results described above are distilled from our analysis of approximately 200 scenarios designed to explore key uncertainties surrounding the impact of current HRHA numbers on ART coverage and TasP implementation. In the S1 File, we describe in detail the results of sensitivity analyses under varying assumptions on HIV transmission probability per sex act, the effect of ART on HIV transmission probability, and ART retention and adherence. The sensitivity analyses in the S1 File show that the findings reported herein are robust to a wide range of assumptions. Here, we present the results of two particularly important sensitivity analyses, evaluating the effects of two phenomena that are directly pertinent to our conclusions: HRHA outmigration and HIV resistance development.

#### HRHA outmigration

**Fig 7:**
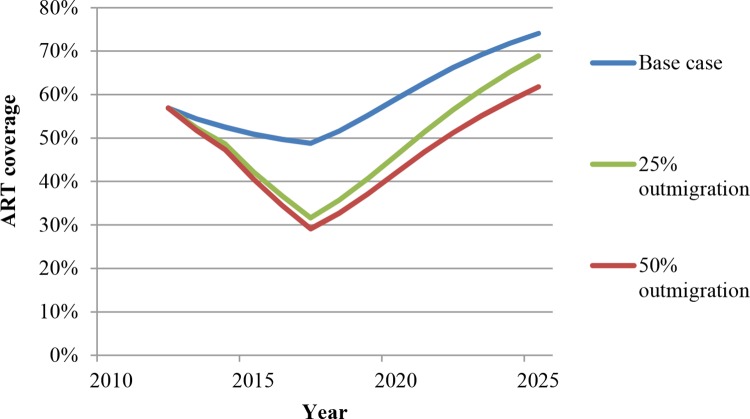
ART coverage in the presence of high levels of outmigration of HRHA.

Recent studies [12, 13] show that health worker outmigration from low-income African countries to high-income countries continues at high levels. We thus examine the effect of two different levels of annual HRHA outmigration (25%, 50%) on our results. As expected, ART coverage is initially reduced in the presence of outmigration, as outmigration implies the HRHA population declines over time until the number of HRHA stabilizes at a new equilibrium (Fig 7). Therefore, the number of new infections and deaths is substantially larger in the presence of higher outmigration (Figs E and F in S1 File). These results emphasize the need to stem the outflow of health workers from Africa. Regarding ART coverage, while the initial reduction in coverage due to outmigration is substantial, the impact of outmigration on ART coverage diminishes over time. This occurs because mortality among sicker patients due to HRHA outmigration outweighs the increase in new infections (Figs E and F in S1 File), causing the epidemic to reach a new steady state.

#### First-line ART resistance

**Fig 8:**
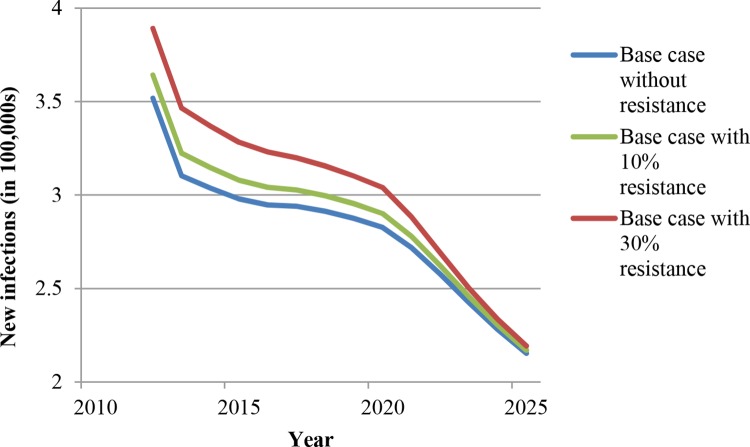
New infections with and without ART resistance (in the absence of second-line ART).

**Fig 9:**
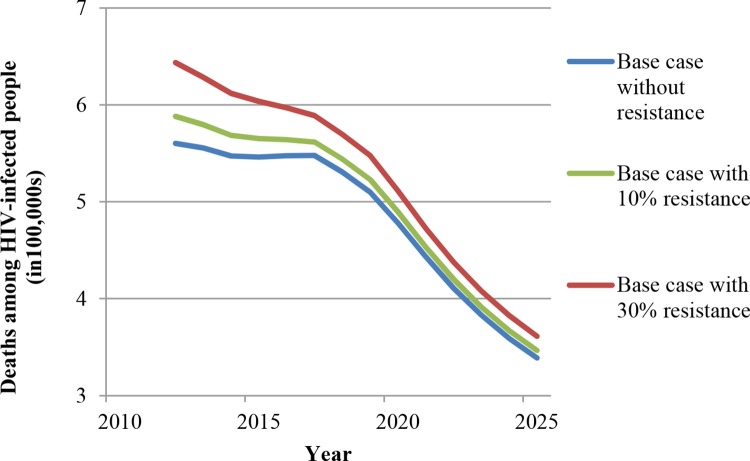
Deaths among HIV-infected people with and without ART resistance (in the absence of second-line ART).

We ran the model with drug resistance levels of 10% and 30% of all patients receiving ART to understand how our results might change. In a recent meta-analysis, 8% of patients on first-line therapy were found to have failed treatment because of drug resistance [63]. Vis-à-vis our assumptions, the 10% and 30% drug resistance levels are thus realistic and conservative, respectively. Two possibilities regarding HIV drug resistance are relevant. The first possibility is that second-line treatment is not available. We have added model runs for this scenario with individuals failing on ART due to resistance, but without effective approaches to address the failure. As expected, relative to the base-case scenario with constant HRHA, the numbers of new infections and deaths increase when 10% of people on ART are resistant to first-line antiretroviral drugs (ARV) and increase even further when 30% of people on ART are resistant to first-line ARV (Figs 8 and 9). However, the declining trajectories in new HIV infections and deaths look similar with these levels of resistance, and the difference between the base-case scenarios with and without resistance declines over time. We have further evaluated the robustness of our findings regarding shifts from ART to TasP under the constant HRHA assumption. While the time patterns and cross-scenario comparisons with resistance remain qualitatively similar to those without resistance, the differences between the “ART only,” the “1/5 resources to TasP,” and the “1/3 resources to TasP” scenarios are compressed by the introduction of resistance (Figs G and H in S1 File for new infections; Figs I and J in S1 File for deaths). These findings make sense because for individuals with resistance, the shift from ART to TasP will not affect outcomes. The larger the proportion of resistant individuals, the less impact the shift from ART to TasP will have. In the extreme case of a completely resistant population, all ART will be ineffective and all different ART strategies will imply the same outcomes.

The second possibility is that second-line treatment is available. Second-line treatment is approximately equally effective as first-line treatment, based on recent empirical results from South Africa [64]. At present in South Africa, viral loads are routinely monitored half-yearly and second-line treatment is available. While some additional effort is required for diagnosis and typology of resistance, these steps are already part of the standard treatment for every patient as laid out in the national South African ART guidelines, with a simple viral load-based clinical management and decision rule and two standard second-line drug regimens (which consist of the drug combination AZT+3TC+LPV/r and TDF+3TC+LPV/r) [65]. Under the current standard in South Africa, the financial outlays increase as a patient is switched from first- to second-line treatment. However, ART effectiveness will not decrease and the health worker time requirements will only increase initially and slightly, because of the highly standardized and guideline-based nature of the second-line decision. Therefore, unlike in the first scenario, resistance in this scenario will not alter the baseline results.

#### Different uptake rates for TasP

**Fig 10:**
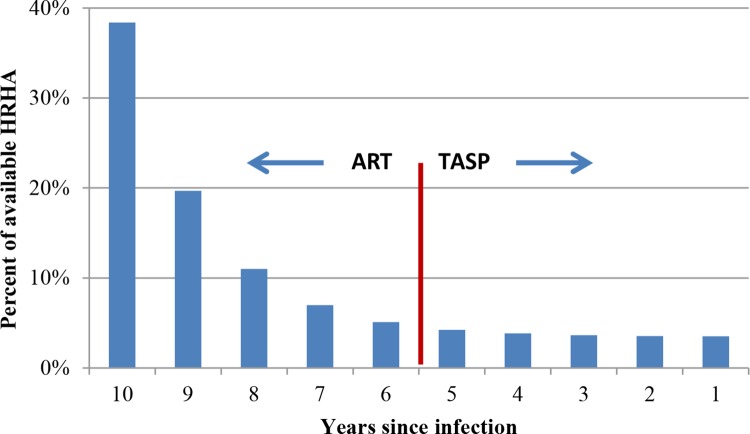
Example scenario for distribution of available HRHA across HIV stages in the model, reflecting an 80/20 distribution of patients on ART/TasP, i.e., 80% of treated patients on ART (CD4 cell count ≤350 μl) and 20% on TasP (CD4 cell count >350 μl).

To assess the impact of shifting resources from ART to TasP, we experimented with two types of distributions of HRHA across different disease stages: proportional and geometric. These distributions reflect how HRHA would be occupied under different uptake rates for TasP. In proportional distribution, HRHA initiate ART such that the number of people on treatment in an HIV-stage is proportional to the total number of HIV-infected people in that disease stage. This disease stage-agnostic ART initiation serves as a basis for comparison with other distribution strategies. In geometric distribution of HRHA, fewer people are initiated on ART such that more patients with advanced disease stages are treated. This allows us to vary the proportion of people who initiate treatment in different disease stages. For example, for ART only (i.e., with no HRHA delivering TasP), one scenario distributes HRHA such that 50% of all who initiate treatment have a CD4 cell count < 200 μl. Similarly, for TasP, scenarios can distribute HRHA such that 20% of those initiated on treatment do so for TasP (Fig 10), or 33% of those initiated receive TasP, with the remaining resources dedicated to ART for disease stages with CD4 cell count <350μl.

## Discussion

Earlier studies indicate that achieving universal ART coverage would not be feasible in many developing countries because of human resources constraints [5, 22]. The reason for this finding was that better survival due to increasing ART coverage made universal coverage a moving goalpost that would become harder to reach with every forward step; each 10% increase in ART coverage would require more human resources than required for the previous 10% increase in ART coverage [5]. This pessimistic assessment was based on the best-available evidence at that time about the mortality-reduction benefits of ART. In this study, we revisit this assessment in light of the evidence about the HIV-prevention effects of ART [31], using South Africa for our country case study. We have also assessed the implications of not expanding the numbers of HRHA on future ART coverage and on health outcomes if TasP is implemented with limited HRHA numbers.

Our analysis of human resource requirements for achieving universal ART coverage yielded three key findings. The first is that our previous understanding of the effect of a fixed quantity of human resources on ART coverage needs to change directionally, in light of the new evidence on the preventive effects of ART. Previous studies found that ART coverage for a fixed quantity of human resources should decline over time. Our results show that the preventive effects of ART are strong enough to increase ART coverage even if the number of human resources remains stationary at current levels. Further, the prediction that increases in HRHA numbers will make universal ART coverage a moving goalpost is no longer true, even if we expect wide variations in transmission probability reduction due to ART.

While our first key finding is optimistic compared with previous conclusions, increased survival due to ART significantly counters the benefits of decreased infections due to ART. The net effect is that increases in ART coverage will be slow if HRHA numbers stagnate at current levels. This raises two policy-related questions: is there a feasible and sustainable path forward for recruiting enough human resources to attain and maintain universal coverage; and what are the implications for health outcomes if we are unable to increase human resources, but implement initiatives such as TasP which will spread these limited resources over a larger pool of people? The latter question has become increasingly important as much of the recent debate around TasP has focused on gathering enough financial resources [66], without considering how the limited availability of human resources, which are much harder to increase, will affect the ambitious targets of providing ART to all HIV-infected people.

Our second key finding is that if the HRHA scale-up in South Africa can continue at the same pace as that of the last few years, and if it can maintain focus on ART delivery rather than on other interventions (such as test-and-treat strategies), South Africa can attain universal ART coverage within five years. Such an HRHA recruitment strategy is sustainable in the long run, as the number of HRHA needed to maintain universal coverage two decades after attaining universal coverage will be roughly comparable to current HRHA numbers. We call such a policy approach the creation of a *surge capacity*, in which an aggressive push is made to recruit additional HRHA for the next five years to deliver ART only and to redeploy these additional HRHA to address other health priorities after attaining universal coverage.

This ART surge-capacity approach will minimize population health losses (mortality and new infections) due to the HIV epidemic as compared with strategies with less aggressive HRHA increases. Quick attainment of universal ART coverage will minimize health losses, and the redeployment of HRHA will become possible through a sharp decrease in HRHA needed after achieving universal coverage. This decrease will occur because the number of people needing ART will eventually stabilize at the natural equilibrium position for the epidemic, in which the number of people becoming newly eligible for ART equals the number of deaths among ART-eligible people. But because the number of people becoming eligible for ART will sharply decline due to the preventive effects of ART, the equilibrium ART-eligible population size will be much lower than it was at the time universal coverage was reached. As the epidemic trends toward this lower equilibrium position, *all HRHA* hired after 2013 for attaining universal coverage in five years can be reassigned to other health priorities 20 years after attaining universal coverage.

Below, we discuss several practical concerns about the ability to create such an HRHA surge capacity. Here we note three pertinent points. First, our recommendation of building a surge capacity for universal ART coverage is practically achievable. Based on multiple literature sources, we estimate that approximately 500–550 additional HRHA (physicians and nurses) were added annually to the pool of ART providers in South Africa over 2010–2012 and approximately 300–350 per year from 2007–2010 [10, 67–73]. Based on these estimates, we assume that if we continue to add 500 HRHA per year for the next five years, the growth required in the number of ART providers will slow in year 6 and become negative from year 7 onward, allowing health planners to start transitioning them out. Even if the creation of this surge capacity is partially successful such that we can only add 250 HRHA per year, Fig R in S1 File shows that the conclusion holds, we can reach universal coverage by 2020 and then initiate a drawdown of HRHA.

Second, in theory, major task shifting or increased efficiency among HRHA may increase the coverage level achievable with the current numbers of HRHA—for instance, through triaging stable ART patients to increase community care and reduce follow-up, freeing up HRHA in clinics to focus on patients requiring active clinical review. However, significant increases of coverage with the current numbers of HRHA will require concomitantly significant levels of efficiency increases or task shifting. Such changes would likely unfold over several years. By that time, a surge capacity of HRHA would have already allowed attainment of universal coverage, at which point efficiency increases or task shifting will only help draw down the HRHA needs faster, allowing for a quicker reassignment to other health needs.

Third, even though it is possible HRHA effectiveness may decline as we approach universal coverage, i.e. the number of HRHA needed to provide ART to 1000 patients may increase because of uneven distribution of clinics and patients, our first two conclusions still hold. ART coverage should rise slowly at current levels of HRHA in the short-term, with a potential slow-down as we near universal coverage; and a surge capacity should allow rapid progress towards universal coverage, with a potential slow-down as we near universal coverage. Sensitivity analyses around alternative ratios of HRHA to patients, using a worst-case assumption (i.e. increasing the number of HRHA needed for 1000 patients even at lower ART levels) confirm this conclusion (Figs P and Q in S1 File).

Our third and last key finding is that if such a surge capacity for ART is not created, but TasP is implemented, a significant threat exists that sicker ART-eligible patients may be crowded out of treatment, compromising the benefits from TasP. Until the point at which the benefits of TasP are fully realized through fewer new infections, the crowding out of these sicker patients can worsen population health outcomes through increased mortality and increased HIV transmission. Recruiting additional HRHA specifically to provide treatment to patients who are not eligible for ART, essentially creating a surge capacity exclusively for TasP, will therefore be worthwhile.

It would be an ironic outcome if TasP, which is intended—and has the potential—to save lives through HIV prevention and treatment, has the unintended consequence of losing lives because insufficient HRHA are available for HIV treatment. One reason for seriously considering this possibility is that TasP implementation can follow one of two broad approaches. It can either be implemented “passively” (i.e., announcing that everyone who is HIV infected is eligible for ART, but not making an extraordinary effort to recruit and retain HIV-infected people in earlier disease stages for TasP), or “actively” (i.e., by making extraordinary efforts to recruit and retain HIV-infected people in earlier disease stages for TasP). In particular, under the “active” approach, unless HRHA are increased commensurately for TasP, we run the risk that sicker HIV patients will not receive needed ART. In the “passive” approach, some have argued that we need not worry as much about crowding out because only patients in late disease stages will demand ART. Of course, in this case, the threat of crowding out may be negligible, but this would be an unfortunate scenario because the expected benefits of TasP would also be forgone.

Another point that deserves mention is that if the crowding-out phenomenon occurs, it will not continue indefinitely, even if TasP is implemented without increasing HRHA capacity. This is because the death of patients who were initiated on ART in later disease stages eventually frees up HRHA for initiating treatment in earlier disease stages, so that fewer people ultimately progress to advanced disease stages and the crowding out stops. However, until then, morbidity and mortality among those who currently need ART will increase. For this reason, we believe the creation of an HRHA surge capacity for TasP should accompany its rollout to accommodate all patients who are added due to TasP. In the long run, this TasP surge capacity can be redeployed similar to the ART surge capacity.

It is further worth noting that if crowding out of sicker patients happens, its negative effects may be larger than we report, since our model does not account for one necessary precondition for TasP: the human resources needed to test large sections of the HIV-uninfected population. By contrast, past ART roll-out has not required increased HIV testing efforts in the general population, because patients start exhibiting other symptoms by the time they are eligible for ART and sicker people tend to seek treatment themselves. For TasP, such testing will be crucial and will require significantly more resources, strengthening our conclusion that if limited human resources are diverted to TasP, either the population health losses may be greater than we have calculated (as scarce health worker capacity will be allocated to supervising and implementing HIV-testing initiatives), or some of the anticipated benefits of TasP may not be realized (as sufficient human resources may not be available for testing). This may prolong the length of time the crowding-out phenomenon persists.

Even if HIV testing were widely available, e.g., through home-based HIV testing [74] or HIV self-testing [75], significant concerns remain about demand for and hence health services outcomes under TasP [76]. In practice, many additional resources beyond testing may be required for creating demand for TasP, e.g., through improving community literacy with respect to HIV treatment [77], peer-based initiatives to increase linkage to HIV care [78], and social marketing campaigns to overcome the deterrent effects of stigma and discrimination [79]. Insofar as these interventions also require human resources, our model is conservative in under-estimating the negative effects of crowding out of sicker patients.

Our findings have two topical implications for global HIV policy. First, donor funding for ART programs in sub-Saharan Africa is likely to decrease or, at best, to stagnate, requiring increasing national funding (or private household) support for ART [38–41, 80]. In the regions, South Africa is furthest advanced in transitioning from external to domestic funding for ART, having embarked on a “PEPFAR transition” in 2012 [81]. Both donors and countries have focused their planning efforts on financial resources needed to manage such transitions, rather than on human and other health system resources needed to achieve successful transitions [41]. The implicit assumption is that health workers working for donor-led programs will be naturally absorbed into national health systems. But this shift in employers may not appeal to some HRHA, who might leave the HIV sector as a result, as some commentators have pointed out [25, 82]. While South Africa is the one exceptional country in the sub-Saharan African region that is already funding the majority of its HIV response from domestic sources [83], evidence on whether the financing transition will have longer-term effects on HRHA in South Africa is still outstanding [39, 42]. Also unclear is whether national programs plan or have the capacity to continue the large-scale recruitment of HRHA seen in the last decade. Given that universal coverage can be achieved within a reasonable time frame, and that HRHA draw-downs can naturally occur after reaching universal coverage, our results suggest it might be better to delay transitions of ART programs for a few years and continue aggressive recruiting of HRHA until the time universal coverage has been reached. The decreasing need for HRHA after that time will afford a more natural and timely opportunity for transitioning these programs.

Second, much of the debate around the push for offering TasP has focused on the health benefits of these changes and the financial resources that might be needed [66]. Policy attention has focused less on what would happen if resource mobilization were to lag behind policy changes, spreading the existing HRHA more thinly. Our results suggest that such inattention can squander the expected health gains from TasP, making considering and planning separately for human resources essential in any roll-out of TasP. Of course, if during the next 5–10 years while a surge capacity for TasP is deployed, disruptive changes to technology occur that radically increase the productivity of health workers (e.g., self-diagnostics, therapeutic cures, effective vaccines), the process of redeployment of health workers to other health domains will speed up, realizing more rapidly the beneficial legacy of HIV treatment for country health systems. Such redeployment of health workers should be feasible both institutionally and technically, because many of the more highly trained health workers who currently work in ART programs previously worked in the general health system. Empirical evidence supports the claim that ART programs, and HRHA, can be successfully integrated into general health systems [84].

In addition, although we have focused on South Africa, the policy insights we discuss should carry over to other hyperendemic countries with generalized epidemics and high ART coverage levels, such as Zimbabwe, Swaziland, Namibia, Lesotho, etc. The countries to which our analysis would not apply are those where the epidemic is still in its initial phases or is in transition. However, in such countries the constraints of HRHA availability are also likely to be less severe (see S1 File for further discussion). Finally, we recognize that several barriers to achieving universal ART coverage exist, including provider-side challenges such as those related to drug access and facilities and equipment capacity, seeker-side issues such as those related to health-seeking behavior, and wider conditions such as societal awareness and social stigma.

### Feasibility and wider benefits of surge capacities

How might a surge capacity be created to achieve universal ART coverage or to implement TasP given the binding HRHA constraints many developing countries face? Current health worker availability and training capacity in sub-Saharan Africa is limited. As an example, South Africa had approximately 38,000 registered medical practitioners and 9,000 registered medical students in 2012 [67]. Current graduation rates are between 1,500 [71] and 1,600 doctors per year [8]. More than half of these graduates intend to study abroad after graduation [85]. Many will emigrate permanently, and most of those who do not will specialize in areas like internal medicine and surgery, rather than working in public-sector ART delivery [85]. In addition, registered practitioners retire from practice or emigrate. Consequently, we estimate an annual net addition of 700–750 per year of registered medical practitioners from 2003–2012, including the number of foreign-qualified doctors that register to practice in South Africa (250–350 per year). How many of the registered practitioners work in private settings is unclear, but estimates have put the number at almost 75% [86]. While some debate exists about the accuracy of such estimates [87], significant expansion of education and training capacity to satisfy medium-term (5–10 years) HRHA needs appears to be infeasible, given the capital and staffing requirements for building medical and nursing schools, the long lead times to set up new institutions, and the long training times required for training doctors and nurses (5–6 years). In addition, increased training is not a sufficient condition for addressing incremental HRHA needs, as expanded capacity will need matching incentives to attract doctors to ART delivery in both the public and private sector.

Still, feasible options do exist for increasing HRHA inflow into ART delivery in the next 5–10 years. Different mixes of such options might be appropriate for different countries, depending on the context. Clearly, countries need to incentivize and aggressively recruit new medical graduates into ART delivery and simultaneously reduce emigration among existing medical practitioners. Policies to recruit new graduates and reduce emigration could include offering “conditional scholarships” for health care education, whereby qualified candidates receive scholarships for study abroad if they enter into a contract to deliver ART in the public sector of their home country for a specific number of years following graduation [88, 89]. Other policy options for reducing emigration include providing training in ART delivery to health workers who are less likely to be internationally mobile for personal or professional reasons [90] and requiring developed countries to adopt so-called ethical recruiting practices, which limit the admission of health workers from developing countries [91], such as the WHO’s *Global Code of Practice on the International Recruitment of Health Personnel* [92].

Further options for increasing HRHA capacity could include increasing the retirement age for nurses in the public sector or actively recruiting already-retired nurses with incentives of better pension and other benefits. Another option could be to attract international graduates to ART programs [93]. South Africa, for instance, has been able to recruit international doctors and nurses to work in rural health centers over the past decade [70]. However, part of its success in attracting foreign workers might have been due to the scale-up of donor-driven ART programs. In general, the number of physicians from developed countries who are willing to work in developing-country ART programs is probably quite limited, and international health workers may not be as effective as locally educated workers because of language difficulties or lack of familiarity with the local health care system.

Finally, better management of existing resources, improved health worker incentive systems, and novel ART delivery approaches could improve the efficiency of HRHA in treating ART patients [94]. For instance, recent evidence from several sub-Saharan African countries seems to suggest that many ART clinics in the region have excess capacity and that better capacity utilization could enhance the efficiency of ART delivery [95]. Novel delivery models–such as allowing patients who have been virally suppressed for prolonged periods of time to visit ART clinics less frequently [96], e.g., annually–could further increase the efficiency of HRHA.

Two additional policy implications flow from the notion of surge capacity. First, given that the additional health workers hired on an expedited basis for ART delivery may eventually be reassigned to provide other health services, making training more broad-based rather than focusing on just HIV might be worthwhile. Second, if the health system can retain the workers recruited specifically to create the surge capacity after reassignment to address other health issues, HIV programs will make a lasting contribution to countries’ health systems and outcomes. This contribution is important because the inability to recruit sufficient health workers for advertised vacancies is among the most significant challenges facing health systems in developing countries [8]. This legacy of a surge-capacity approach could speed up the achievement of “convergence” envisioned by the Lancet Commission on Global Health [19], i.e., where the size of the gap in preventable mortality and infectious disease burdens between high-mortality and low-mortality countries can be significantly reduced in a single generation.

One caveat about our analysis and recommendations is that we consider the allocation of human resources among alternative HIV interventions, but not across other health priorities. The net impact on health systems of recruiting increasing numbers of health workers into ART programs is under debate [97–100]. On the one hand, increasing the numbers of HRHA may to adversely affect other health sector functions. For instance, some evidence suggests that the HIV response is distorting health worker allocations to other health sector issues [17] (e.g., large numbers of health workers working on HIV in low HIV-prevalence countries). Whether or not such inappropriate choices have happened in many countries is unclear. But the potential influences of aggressive HRHA scale-up both on in-country resources and on the emigration choices of neighboring resource-limited countries should be empirically studied and carefully considered in health policy formulation. On the other hand, utilizing increasingly large numbers of health workers for ART may lead to decreasing population need for other health care, because ART avoids the substantive health burdens of the later stages of HIV disease, which commonly lead to expensive treatment and hospitalization [101]. These broader health systems implications further emphasize that it is important to consider HRHA surge capacity in the broader context of strategic planning for human resources for health, including long-term health worker education and emigration policies [102, 103].

As many countries in sub-Saharan Africa are currently moving to TasP policies, or considering such policy changes, how HRHA surge capacity can be achieved is becoming an urgent question. We have demonstrated here the need for surge capacity to ensure that the full health benefits of TasP policies can be realized. Future research–both empirical and predictive–needs to establish whether TasP policies can become reality (e.g., how much demand-inducement will be needed as an accompanying intervention) and how the additional capacity required for effective TasP can be created in the short and in the longer term.

## Supporting Information

S1 File Supporting Information. Human resources for treating HIV/AIDS: Are the preventive effects of antiretroviral treatment a game changer?
(DOCX)
