# Supplementary material for: Human Resources for Treating HIV/AIDS: Are the Preventive Effects of Antiretroviral Treatment a Game Changer?
Source: PLoS One. 2016 Oct 7;11(10):e0163960. doi: 10.1371/journal.pone.0163960 (PMC5055321; doi:10.1371/journal.pone.0163960)
Supplement: S1 File — Human resources for treating HIV/AIDS: Are the preventive effects of antiretroviral treatment a game changer? (DOCX) [file pone.0163960.s001.docx]

Supporting Information

Human resources for treating HIV/AIDS: Are the preventive effects of antiretroviral treatment a game changer?

**Till Bärnighausen,^1,2,3^ David E. Bloom,^1^ and Salal Humair^4^**

^1^ Department of Global Health and Population, Harvard T.H. Chan School of Public Health, Boston, Massachusetts, United States of America; ^2^ Africa Health Research Institute (AHRI), Mtubatuba, KwaZulu Natal, South Africa; ^2^ Institute of Public Health, University of Heidelberg, Heidelberg, Germany; ^4^ Amazon.com, Inc., Seattle, Washington, United States of America

Introduction

This supporting information provides additional detail on the model used in the paper “Human resources for treating HIV/AIDS: Are the preventive effects of antiretroviral treatment a game changer?” including a discussion of the assumptions in the model, its mathematical formulation, and the data sources used for the analysis.

The paper uses a mathematical model to study how the HIV-prevention benefits of ART affect human resource needs in countries with generalized epidemics, using South Africa as an example. Several considerations motivated the choice of South Africa. First, the country has the largest number of HIV-infected individuals in the world (6.4 million [1]), the largest number of individuals on ART (almost 2.1 million [2]), and the largest number of new HIV infections per year (400,000 [1]). Second, South Africa is within reach of achieving universal ART coverage under current ART eligibility thresholds (CD4 cell count <350µl) if the impressive expansion of its ART programs as over the last few years continues. But the country is likely to see a surge in need for antiretroviral treatment if treatment-as-prevention (TasP) programs are implemented. Third, South Africa is poised for potentially major changes in ART programs, as the United States Presidential Emergency Program for AIDS Relief (PEPFAR) is reducing and redirecting its support for South African HIV programs [3, 4]. Fourth, as the switch of PEPFAR occurs from 2012–2017, the current HIV financing plan envisages an initial increase followed by a flat-lining of total resources by 2017 [3]. In this environment, policy insights, such as the *surge-capacity* approach our study suggests and the note of caution about the potential of *crowding out* sicker patients as treatment as prevention (TasP) is implemented without adequate expansion of human resources, assume larger importance. Finally, for both health worker staffing patterns and the HIV epidemic, good-quality empirical data are available for South Africa.

Materials and methods

The model we use in this study advances the model from [5] referred to as the BBH (Bärnighausen, Bloom, and Humair) model. However, it is substantially different because of the addition of adherence and retention, the use of fixed resources (rather than a fraction of population covered), and the ability to distribute these fixed resources flexibly among different disease stages.

Several models have been independently developed to understand the evolution of the HIV epidemic and to project the impact of different HIV-related interventions [6-14]. These models have different strengths depending on the questions they were designed to answer (in general individual models cannot answer every question other models taken together can), have very different data requirements, and for the most part also require statistical calibration by fitting a curve to the observed history of the disease. To answer our specific research questions regarding the effect of alternative health workforce resource allocations for HIV treatment, we needed to develop a new model of the HIV epidemic. Standard models of the HIV epidemic assume that the rate of change of population sizes in different disease stages is proportional to population sizes. To project the epidemic trajectory under alternative distributions of a fixed number of health workers across patients in different disease stages, we needed a model in which the rate of change of a population size (in a disease stage) is not proportional to the population size, but to the minimum of the available resources and the population size. To achieve this, we developed a model of the HIV epidemic based on causal theory: in closed-form equations we mathematically specify hypothesized causal relationships that connect variables with well-understood biological and behavioral definitions. The model that resulted has the advantage of a parsimonious set of assumptions and does not require fitting a curve to HIV-epidemic history. In fact, using similar parameters as other models (such as transmission probabilities, mortality rates, etc.) from published literature, but not modifying them so as to fit a curve, the model produces epidemic outcomes such as HIV incidence, prevalence, etc., that coincide with the historical epidemic trajectory for generalized epidemics.

The assumptions underlying the BBH model are detailed in [5]. Here, we only comment on four that bear on our current study. First, because ART is highly effective in stopping the progression of HIV, we assume that a person receiving ART does not move to the next stage of the disease until he or she stops receiving ART. For instance, if a treatment-naive person were to start receiving ART, his or her CD4 cell count would remain static, until suffering mortality or stopping ART. This assumption is conservative in the sense that it may underestimate the preventive benefits of ART, because in practice ART usually results in a recovery of CD4 cell count. However, we believe the underestimate would be marginal because the mortality and the transmission probability for a person receiving ART is roughly comparable across HIV disease stages, so it does not make a material difference if the CD4 cell count of a person on ART is left at the ART-initiation stage or raised.

Second, we model nonretention and nonadherence as either fully compliant or completely noncompliant. Thus a person not retained on ART in a period does not get any benefits in terms of transmission probability reduction and mortality reduction for that period, and progresses to the next stage of the disease in the next period switching her status to not receiving ART. Similarly, among the people retained on ART in a period, a person who is nonadherent receives no benefits of ART and progresses to the next stage of the disease in the next period while maintaining her receiving ART status. These assumptions are again conservative in the sense that they may underestimate the benefits of ART. However, introduction of a more graded adherence response, where benefits of ART would depend on the degree of ART adherence, would introduce more complication into the model and increase data requirements, some of which are not available (e.g., the degree of benefits of ART realized under different adherence levels).

Third, the manner in which we model nonretention and nonadherence (as yearly rates) implies that the times to individuals changing their retention and adherence behavior are random variables with a geometric probability distribution. If we were to impose non-geometric distributions on individuals changing their behavior, it would force us to hypothesize a specific non-geometric distribution, since empirical data on the probability distribution of times people spend being non-adherent or during non-retention are not available. Further, while making the model much more complicated (requiring the length of time people have been non-adherent to be carried in the model state), it would make marginal contributions to the population-level results as our sensitivity analyses for higher nonretention and nonadherence rates of 30% and 50% show (e.g., Fig 2 in the paper and Fig O).

Fourth and finally, we assume that condom usage rates and circumcision levels for South Africa will remain constant at current levels for the foreseeable future. Clearly that may not be the case, as both condom and circumcision rates will likely increase, but the intent of this model is to compare the impact of HRHA-related policies, and these assumptions serve as a reasonable baseline with which to do so. In fact, if coverage rates for these other intervention increase, some of the conclusions from our analyses may become stronger.

The results of our analysis are robust for South Africa in two senses. They are comparable to the predictions from several other models for comparable interventions, including TasP [12, 14]. Further, our results remain strong even when compared against an overtly optimistic case for South Africa that should have made our results appear weaker, i.e., when HIV incidence in South Africa is already declining due to increased uptake of prevention interventions other than ART (e.g., condom usage, circumcision, etc.). Finally, our key insights can likely be applied to other countries with generalized and stable epidemics where the primary mode of HIV transmission is heterosexual. The reason for this generalizability is that our model involves a minimal set of assumptions. At its most essential level, the model is a distribution of a male and female population into uninfected and HIV-infected states differentiated by CD4 cell count levels. The only other assumption is that of a random selection of partners between the male and female populations. For a country like South Africa, and indeed for countries with reasonably sized populations and generalized epidemics, the law of averages is likely to produce what looks like a random selection of partners at a national level, even though it may look very different at a regional and localized level, or when focusing on specialized populations or specific risk-behavior groups.

Our model captures the first-order effects (e.g., mortality, transmission probability reduction, etc.) of policy choices related to the human resources scale-up for HIV treatment. Although adding complicating factors such as drug resistance, etc., is always possible, their effects would likely be second-order. Consider drug resistance for example. The 2012 WHO report on HIV Drug Resistance [15] suggests the prevalence of drug resistance peaked in low- and middle-income countries in about 2009 at 6.6%, and the relatively low levels of drug resistance that did occur were controllable through prompt switching to second-line therapy after first-line failure. Hence the effects of drug resistance appear to be distinctly second-order relative to those of mortality and transmission probability reduction due to ART. Further, which of the second-order effects will actually dominate the other in the long run is not clear. For instance, recent reports [16, 17] suggest the possibility that HIV might be becoming less virulent, in part because of the increased use of ART. Such an effect, if it strengthens, might partially compensate for the negative second-order effects, such as those feared because of increasing drug resistance due to ART.

Our model has some limitations. First, we assume that the epidemic is in its stable phase, i.e., prevalence is beyond the typical S-shaped inflection point seen in some emerging epidemics, and as a result our key insight may not apply to epidemics that have not reached stability. Two pragmatic reasons for this assumption are that an epidemic in its early stages is of less interest because the HRHA needs are less and, for most countries with generalized epidemics, the epidemic is likely in a stable phase. Second, we model a closed system, a good approximation to national systems where the infections predominantly arise internally (as opposed to coming from outside). This system would not be a good approximation if there was reason to believe that a significant number of HIV infections arise through exogenous inflows (e.g., international migrants or tourists bringing in infections, or in a localized setting such as sex workers frequented by mobile clients such as truckers). It would also not be a good approximation if a significant number of new infections were to arise through pathways not considered in our model, such as injecting drug use or contaminated blood supplies.

Mathematical formulation

Notation

$t$: Time period, $t=0,\cdots,\infty$.

$j$: Indexes the number of years since infection for an ART-naive population. These years represent declining CD4 cell count categories, and for a population receiving ART and adherent to ART, the CD4 cell count decline stops at the time of initiation on ART. We consider $j$ to be bounded, as $0\leq j\leq U$, where $U$ is an upper bound, with the upper and lower bounds having special interpretations: $j=0$ denotes the uninfected population, and $j=U$ denotes population that has been infected $U$ years or longer.

$k$: Indexes a subdivision of the population in the $j$th year of its infection, $k\geq0$. For men, $k=1,\ldots,4$ ($1=$ uncircumcised not receiving ART, $2=$ uncircumcised receiving ART, $3=$ circumcised not receiving ART, $4=$ circumcised receiving ART) in each year $j$. For women, $k=1, 2$ ($1=$ not receiving ART, $2=$ receiving ART).

Populations age 15 or older

$L_{m}$: The number of years from the start of HIV infection to the time CD4 <200 µl for ART-naïve men.

$L_{w}$: The number of years from the start of HIV infection to the time CD4 <200 µl for ART-naïve women.

$E_{m}$: The number of years from start of infection to when men are eligible to receive ART for treatment according to WHO guidelines.

$E_{w}$: The number of years from start of infection to when women are eligible to receive ART for treatment according to WHO guidelines.

$M_{t}^{jk}$: Number of men at the beginning of period $t$ who are in the $j$th year of their infection and belong to the $k$th subdivision within that year. $j=0, 1,\cdots,L_{m}+1$, where $j=0$ represents HIV-uninfected men and $j=L_{m}+1$ represents men infected $L_{m}+1$ years or longer. As above, $k=1,\ldots,4$, with $1=$ uncircumcised not receiving ART, $2=$ uncircumcised receiving ART, $3=$ circumcised not receiving ART, $4=$ circumcised receiving ART. $M_{t}^{02}=M_{t}^{04}=0$ because we only allow ART for infected men.

$W_{t}^{jk}$: Number of women at the beginning of period $t$ who are in the $j$th year of their infection belong to the $k$th subdivision within that year. $j=0, 1,\cdots,L_{w}+1,$ where $j=0$ represents HIV-uninfected women and $j=L_{w}+1$ represents women infected $L_{w}+1$ or longer. As above, $k=1, 2,$ with $1=$ not receiving ART, $2=$ receiving ART). $W_{t}^{02}=0$ because we only allow ART for infected women.

Incidence and mortality

$\alpha_{m}^{0},\alpha_{m}^{1}$: HIV-uninfected and HIV-infected men exogenously arriving each year into the HIV-uninfected pool and HIV-infected pool of men who are in the first year of their infection.

$\alpha_{w}^{0},\alpha_{w}^{1}$: HIV-uninfected and HIV-infected women exogenously arriving each year into the HIV-uninfected pool and HIV-infected pool of women who are in the first year of their infection.

$m_{m}^{jk}$: Mortality rate for men in the $j$th year of infection and the $k$th subdivision in that year.

$m_{w}^{jk}$: Mortality rate for women in the $j$th year of infection and the $k$th subdivision in that year.

Coverage of other interventions

$C_{cir}$: The proportion of the initial male population that is circumcised and the fraction of exogenously arriving 15+ year olds who are circumcised.

$C_{con}$: Condom usage, defined as the proportion of annual sex acts with a partner protected by condom use.

Sexual behavior

$p_{m}^{jk}$: The probability that an HIV-infected man in the $j$th year of his infection and the $k$th subdivision of that year infects his partner in a single sex act.

$p_{w}^{jk}$: The probability that an HIV-infected woman in the $j$th year of her infection and the $k$th subdivision of that year infects her partner in a single sex act.

$p_{c}$: The percentage reduction in HIV-acquisition probability per sex act for men. For a circumcised man with an HIV-infected partner who has a transmission probability per sex act of $x$, the HIV acquisition probability per sex act is $p_{c}x$.

$N_{m},P_{m}(n)$: The random variable representing the number of sexual partners a man can have in a year and the probability that the number of these partners is $n=0,\cdots,n_{m}^{\text{max}}$.

$N_{w},P_{w}(n)$: The random variable representing the number of sexual partners a woman can have in a year and the probability that the number of these partners is $n=0,\cdots,n_{w}^{\text{max}}$.

$A_{m}$: The average number of unprotected annual sex acts a man has with one partner (total sex acts per year multiplied by ${(1-C}_{con})$).

$A_{w}$: The average number of unprotected annual sex acts a woman has with one partner (total sex acts per year multiplied by ${(1-C}_{con})$).

Treatment behavior

$\beta_{m}^{jk}$: The fraction of HIV-infected men in disease stage $j$ and subdivision $k$ receiving ART who are not retained on ART for more than a year.

$\beta_{w}^{jk}$: The fraction of HIV-infected women in disease stage $j$ and subdivision $k$ receiving ART who are not retained on ART for more than a year.

$\gamma_{m}^{jk}$: The fraction of men in disease stage $j$ and subdivision $k$ retained on ART who are nonadherent to treatment. For convenience, we define $\gamma_{m}^{j1}=\gamma_{m}^{j3}=1$ (i.e., all men not receiving ART are by definition nonadherent) and let $0\leq\gamma_{m}^{j2},\gamma_{m}^{j4}\leq1,$(i.e., all men receiving ART can have some fraction that are nonadherent).

$\gamma_{w}^{jk}$: The fraction of women in disease stage $j$ and subdivision $k$ retained on ART who are nonadherent to treatment. For convenience, we define $\gamma_{m}^{j1}=1$ (i.e., all women not receiving ART are by definition nonadherent) and let $0\leq\gamma_{m}^{j2}\leq1,$ (i.e., all women receiving ART can have some fraction that are nonadherent).

Human resources

$H_{t}$: Total human resources available for providing ART in period $t$.

$\varphi$: Number of patients that can be provided ART in one year by a one unit of human resources.

${HM}_{t}$: Total human resources available for providing ART to men in period $t$.

${HW}_{t}$: Total human resources available for providing ART to women in period $t$.

${FHM}_{t}^{j}$: Fraction of human resources available for providing ART to men in period $t$ that are used to provide ART to men in stage $j$ of the disease.

${FHW}_{t}^{j}$: Fraction of human resources available for providing ART to women in period $t$ that are used to provide ART to women in stage $j$ of the disease.

Formulation

Assuming all quantities except time are continuous, the model formulation is as follows. Initial values at $t=0$ for all quantities indexed by $t$ are given. Events are assumed to be independent over time and are ordered in the following manner in each period: at the beginning of the period, the current populations are used to calculate the new sexually transmitted infections that are expected over the year; loss through deaths occurs at the end of the period and individuals are removed from the population; and then new arrivals to the populations occur and the latency populations are moved forward in time, transitioning the model to the next period.

Sexually transmitted infections

Each HIV-uninfected individual selects a partner at random from the population of opposite-sex individuals each year, independently of any partners he or she has chosen before or will choose in the future. He or she has a given average number of sex acts with this partner over the year. If the chosen partner is HIV infected, each sex act has a given probability of him or her acquiring the infection, independently from any other sex acts they have. With a certain other probability, the same person can choose multiple partners and repeat the same process with them. In our infection model, we are also not considering the possibility of HIV superinfection through acquisition of a second, heterologous HIV strain after an initial primary infection.

Then the probability ${PNIM}_{t}$, that an uncircumcised man does not get infected from a single partner in the year, and the total probability of him getting infected during the year, $P{IM}_{t}$, are (where $\bar{W_{t}}=\sum_{j=0}^{L_{w}+1} \sum_{k=1}^{2} W_{t}^{jk}$ and $p_{w}^{j,k}$ is an alternative way of writing $p_{w}^{jk}$ to distinguish the superscripts $j$ and $k$):

$${PNIM}_{t}=\sum_{k=1}^{2} \frac{W_{t}^{0k}}{\overline{W}_{t}}+\sum_{j=1}^{L_{w}+1} \frac{W_{t}^{j1}}{\overline{W}_{t}}\left( 1-p_{w}^{j1} \right)^{A_{m}}+\sum_{j=1}^{L_{w}+1} \frac{W_{t}^{j2}}{\overline{W}_{t}}\left[ {\gamma_{w}^{j2}\left( 1-p_{w}^{j1} \right)}^{A_{m}}+\left( 1-\gamma_{w}^{j2} \right)\left( 1-p_{w}^{j2} \right)^{A_{m}} \right],$$

$$P{IM}_{t}=1-\sum_{n=1}^{n_{m}^{\text{max}}} P_{m}(n)\left( {PNIM}_{t} \right)^{n}.$$

These expressions assert that if the female partner of a man is taking ART but is nonadherent with probability $\gamma_{w}^{j2}$, her HIV-transmission probability is the same as if she were not taking ART. This is clearly a worst-case assumption, but given the lack of data on how much nonadherence may affect the transmission probability is a practical and conservative assumption.

The corresponding probability that a circumcised male does not get infected from a single partner in the year, ${PNICM}_{t}$, and the total probability of him getting infected during the year, $P{ICM}_{t}$, are expressed below. Again, these probabilities account for the effects of a partner’s nonadherence.

$${PNICM}_{t}=\sum_{k=1}^{2} \frac{W_{t}^{0k}}{\overline{W}_{t}}+\sum_{j=1}^{L_{w}+1} \frac{W_{t}^{j1}}{\overline{W}_{t}}\left( 1-{p_{c}p}_{w}^{j1} \right)^{A_{m}}+\sum_{j=1}^{L_{w}+1} \frac{W_{t}^{j2}}{\overline{W}_{t}}\left[ {\gamma_{w}^{j2}\left( 1-{p_{c}p}_{w}^{j1} \right)}^{A_{m}}+\left( 1-\gamma_{w}^{j2} \right)\left( 1-{p_{c}p}_{w}^{j2} \right)^{A_{m}} \right],$$

$$P{ICM}_{t}=1-\sum_{n=1}^{n_{m}^{\text{max}}} P_{m}(n)\left( {PNICM}_{t} \right)^{n}.$$

The probability ${PNIW}_{t}$, that a woman does not get infected from a single partner in the year, and her total probability of getting infected during the year, $P{IW}_{t}$, are as follows ($\bar{M_{t}}=\sum_{j=0}^{L_{m}+1} \sum_{k=1}^{4} M_{t}^{jk})$:

$${PNIW}_{t}=\sum_{k=1}^{4} \frac{M_{t}^{0k}}{\overline{M}_{t}}+\sum_{k=\left\{ 1,3 \right\}} \sum_{j=1}^{L_{m}+1} \frac{M_{t}^{jk}}{\overline{M}_{t}}\left( 1-p_{m}^{jk} \right)^{A_{w}}+\sum_{k=\left\{ 2,4 \right\}} \sum_{j=1}^{L_{m}+1} \frac{M_{t}^{jk}}{\overline{M}_{t}}\left[ {\gamma_{m}^{jk}\left( 1-p_{m}^{j,k-1} \right)}^{A_{w}}+\left( 1-\gamma_{m}^{jk} \right)\left( 1-p_{m}^{jk} \right)^{A_{w}} \right],$$

$$P{IW}_{t}=1-\sum_{n=1}^{n_{w}^{\text{max}}} P_{w}(n)\left( {PNIW}_{t} \right)^{n}.$$

Next state for HIV-uninfected population

Given the initial state at $t=0$ and the probabilities of sexual infection under the ordering of events described at the beginning of the section, the state evolution for $t=1,\cdots,\infty$ is as follows. The transitions for men for $j=0$ are

$M_{t+1}^{01}=\left( 1-m_{m}^{01} \right)\left( 1-{PIM}_{t} \right)M_{t}^{01}+\alpha_{m}^{0}(1-C_{cir})$,

$M_{t+1}^{02}=0$,

$M_{t+1}^{03}=\left( 1-m_{m}^{03} \right)\left( 1-{PICM}_{t} \right)M_{t}^{03}+\alpha_{m}^{0}C_{cir}$,

$M_{t+1}^{04}=0$.

For uninfected women, $j=0$, they are

$W_{t+1}^{01}=\left( 1-m_{w}^{01} \right)\left( 1-{PIW}_{t} \right)W_{t}^{01}+\alpha_{w}^{0}$,

$W_{t+1}^{02}=0$.

Interim next state for HIV-infected population

Given the state at $t$, define the following interim states for men in period $t+1$ for $j=0,\cdots,L_{m}$. The interim states will allow us to express the next state for men compactly, after we describe the allocation of human resources. To make the expressions below more compact, we denote $m_{m}^{jk}=1-m_{m}^{jk}, \beta_{m}^{jk}=1-\beta_{m}^{jk}$, and $\gamma_{m}^{jk}=1-\gamma_{m}^{jk}$.

$${M^{'}}_{t+1}^{(j+1)1}=\left\{ \begin{aligned} \begin{aligned} m_{m}^{j1}M_{t}^{j1}{PIM}_{t}+\alpha_{m}^{1}\left( 1-C_{cir} \right), \text{if} j=0, \\ m_{m}^{j1}M_{t}^{j1}+ m_{m}^{j1}\beta_{m}^{j2}M_{t}^{j2}, \text{if} 0<j<L_{m}, \end{aligned} \\ m_{m}^{j1}M_{t}^{j1}+ m_{m}^{j1}\beta_{m}^{j2}M_{t}^{j2}+m_{m}^{j1}M_{t}^{\left( j+1 \right)1}+m_{m}^{(j+1)1}\beta_{m}^{\left( j+1 \right)2}M_{t}^{(j+1)2}, \text{if} j=L_{m}, \end{aligned} \right.$$

${M^{'}}_{t+1}^{(j+1)2}= \left\{ \begin{aligned} m_{m}^{(j+1)2}\gamma_{m}^{(j+1)2}{\beta_{m}^{(j+1)2}M}_{t}^{(j+1)2},\text{ }\text{ }\text{if }j=0, \\ \begin{aligned} m_{m}^{j1}\beta_{m}^{j2}\gamma_{m}^{j2}M_{t}^{j2}+m_{m}^{(j+1)2}\gamma_{m}^{(j+1)2}{\beta_{m}^{(j+1)2}M}_{t}^{(j+1)2},\text{ }\text{ }\text{ }\text{ }\text{i}\text{f}\text{ }0<j<L_{m}, \\ m_{m}^{j1}\beta_{m}^{j2}\gamma_{m}^{j2}M_{t}^{j2}+\left( m_{m}^{(j+1)2}\gamma_{m}^{(j+1)2}\beta_{m}^{(j+1)2}+m_{m}^{(j+1)1}\beta_{m}^{\left( j+1 \right)2}\gamma_{m}^{\left( j+1 \right)2} \right)M_{t}^{(j+1)2}, \text{if}\text{ }j=L_{m}, \end{aligned} \end{aligned} \right.$

$${M^{'}}_{t+1}^{(j+1)3}=\left\{ \begin{aligned} \begin{aligned} m_{m}^{j3}M_{t}^{j3}{PICM}_{t}+\alpha_{m}^{1}C_{cir}, \text{if} j=0, \\ m_{m}^{j3}M_{t}^{j3}+ m_{m}^{j3}{\beta_{m}^{j4}M}_{t}^{j4}, \text{if} 0<j<L_{m}, \end{aligned} \\ m_{m}^{j3}M_{t}^{j3}+ m_{m}^{j3}{\beta_{m}^{j4}M}_{t}^{j4}+m_{m}^{j3}M_{t}^{\left( j+1 \right)3}+m_{m}^{\left( j+1 \right)3}\beta_{m}^{\left( j+1 \right)4}M_{t}^{\left( j+1 \right)4}, \text{if} j=L_{m}, \end{aligned} \right.$$

$${M^{'}}_{t+1}^{(j+1)4}=\left\{ \begin{aligned} m_{m}^{\left( j+1 \right)4}\gamma_{m}^{(j+1)4} \beta_{m}^{\left( j+1 \right)4}M_{t}^{\left( j+1 \right)4},\text{ }\text{ }\text{ }\text{ }\text{ if }j=0, \\ \begin{aligned} {m_{m}^{j3}\beta_{m}^{j4}\gamma_{m}^{j4}M_{t}^{j4}+m}_{m}^{(j+1)4}{\gamma_{m}^{(j+1)4} \beta_{m}^{(j+1)4}M}_{t}^{(j+1)4},\text{ }\text{ }\text{ if }0<j<L_{m}, \\ m_{m}^{j3}\beta_{m}^{j4}\gamma_{m}^{j4}M_{t}^{j4}+\left( m_{m}^{\left( j+1 \right)4}\gamma_{m}^{(j+1)4} \beta_{m}^{\left( j+1 \right)4}+m_{m}^{\left( j+1 \right)3}\beta_{m}^{\left( j+1 \right)4}\gamma_{m}^{\left( j+1 \right)4} \right)M_{t}^{\left( j+1 \right)4},\text{ }\text{ }\text{if }j=L_{m}. \end{aligned} \end{aligned} \right.$$

The interim states above include the effects of nonadherence to ART and nonretention in ART programs. They assert that people who are not retained on ART move to the next-worse disease stage at the end of the year, switching their status to those not receiving ART. In addition, those who are retained but are not adherent to ART do not gain benefits of treatment, so even though they may be considered on treatment, their disease also progresses naturally to a worse state, although they retain their status as those receiving ART. Both non-retained and non-adherent people suffer mortality as if they were not receiving ART.

For women, interim states are defined analogously. Given For $j=0,\cdots,L_{w}$, we define interim states for women as for men, incorporating the effects of nonadherence and nonretention by women.

$${W^{'}}_{t+1}^{(j+1)1}=\left\{ \begin{aligned} \begin{aligned} m_{w}^{j1}W_{t}^{j1}{PIW}_{t}+\alpha_{w}^{1}, \text{if} j=0, \\ m_{w}^{j1}W_{t}^{j1}+m_{w}^{j1}\beta_{w}^{j2}W_{t}^{j2}, \text{if} 0<j<L_{w}, \end{aligned} \\ m_{w}^{j1}W_{t}^{j1}+m_{w}^{j1}\beta_{w}^{j2}W_{t}^{j2}+m_{w}^{j1}W_{t}^{\left( j+1 \right)1}+m_{w}^{\left( j+1 \right)1}\beta_{w}^{\left( j+1 \right)2}W_{t}^{\left( j+1 \right)2}\text{, }\text{if} j=L_{w}, \end{aligned} \right.$$

$${W^{'}}_{t+1}^{\left( j+1 \right)2}=\left\{ \begin{aligned} m_{w}^{\left( j+1 \right)2}{\gamma_{w}^{\left( j+1 \right)2}\beta_{w}^{\left( j+1 \right)2}W}_{t}^{\left( j+1 \right)2},\text{ }\text{ }\text{if }j=0, \\ \begin{aligned} m_{w}^{j1}\beta_{w}^{j2}\gamma_{w}^{j2}W_{t}^{j2}+m_{w}^{\left( j+1 \right)2}{\gamma_{w}^{\left( j+1 \right)2}\beta_{w}^{\left( j+1 \right)2}W}_{t}^{\left( j+1 \right)2},\text{ }\text{ }\text{if }0<j<L_{w}, \\ m_{w}^{j1}\beta_{w}^{j2}\gamma_{w}^{j2}W_{t}^{j2}+\left( m_{w}^{\left( j+1 \right)2}\gamma_{w}^{\left( j+1 \right)2}\beta_{w}^{\left( j+1 \right)2}+m_{w}^{\left( j+1 \right)1}\beta_{w}^{\left( j+1 \right)2}\gamma_{w}^{\left( j+1 \right)2} \right)W_{t}^{\left( j+1 \right)2},\text{ }\text{ }\text{if }j=L_{w}. \end{aligned} \end{aligned} \right.$$

Distribution of human resources across disease stages

For proportional distribution of HRHA across disease stages, reflecting first come first serve and equal demand for treatment from all ART-eligible disease stages, we let ${FHM}_{t}^{j}$ and ${FHW}_{t}^{j}$ be the proportions of ART-eligible populations in the $j$th disease stage. So for instance, ${FHM}_{t}^{j}={\sum_{k=1}^{4} M_{t}^{jk}}/{\sum_{i=E_{m}+1}^{L_{m}+1} \sum_{k=1}^{4} M_{t}^{ik}}$, for $j=E_{m}+1,\cdots,L_{m}+1$ and $0$ otherwise. Similarly ${FHW}_{t}^{j}={\sum_{k=1}^{2} W_{t}^{jk}}/{\sum_{i=E_{w}+1}^{L_{w}+1} \sum_{k=1}^{2} W_{t}^{ik}}$ for $j=E_{w}+1,\cdots,L_{w}+1$ and $0$ otherwise.

When human resources are to be split disproportionately among different disease stages (as we assume for TasP), we use a parameterized function to distribute resources. The essential idea is to distribute fewer resources to earlier disease stages to reflect the potentially lower demand for ART for less sick patients. To do so, we define a function parameterized by three numbers, $x_{min}, x_{max}, \alpha$. The form of the function is $f_{j}=x_{min}+\alpha^{L_{w}+1-j}(x_{max}-x_{min})$. Then ${FHW}_{t}^{j}={f_{j}}/{\sum_{k=1}^{L_{w}+1} f_{k}}$ and ${FHM}_{t}^{j}={f_{j}}/{\sum_{k=1}^{L_{m}+1} f_{k}}$. It can be verified that by setting $x_{max}=4, x_{min}=1, \alpha=0.53$, around one-third of HRHA will be allocated to TasP and the rest to ART. Similarly, by setting $x_{max}=11, x_{min}=1, \alpha=0.46$, roughly one-fifth of the human resources will be distributed to TasP.

Allocation of human resources among men, women and disease stages

If human resources provided are split proportionally between men and women, then at time $t$,

$${HM}_{t}=\frac{\overline{M}_{t}-\sum_{k=1}^{4} M_{t}^{0k}}{\overline{M}_{t}+\overline{W}_{t}-\sum_{k=1}^{4} M_{t}^{0k}-\sum_{k=1}^{2} W_{t}^{0k}}H_{t},$$

$${HW}_{t}=\frac{\overline{W}_{t}-\sum_{k=1}^{2} W_{t}^{0k}}{\overline{M}_{t}+\overline{W}_{t}-\sum_{k=1}^{4} M_{t}^{0k}-\sum_{k=1}^{2} W_{t}^{0k}}H_{t}.$$

We can then express the distribution of these human resources across disease stages $j=1,\cdots,L_{m}+1$ for men as ${HM}_{t}{FHM}_{t}^{j}$. For each disease stage $j$, however, we further need to allocate human resources to those serving circumcised men and those serving uncircumcised men, for purposes of tracking further state transitions. These sub-allocations are obtained using the interim state for men described previously. Thus the number of human resources providing ART to uncircumcised men is

$${HM}_{t}^{j2}=\frac{{M^{'}}_{t}^{j1}+{M^{'}}_{t}^{j2}}{\sum_{k=1}^{4} {M^{'}}_{t}^{jk}}{HM}_{t}{FHM}_{t}^{j},$$

And the number providing ART to circumcised men is

$${HM}_{t}^{j4}=\frac{{M^{'}}_{t}^{j3}+{M^{'}}_{t}^{j4}}{\sum_{k=1}^{4} {M^{'}}_{t}^{jk}}{HM}_{t}{FHM}_{t}^{j}.$$

For women, we do not need to further allocate human resources and can simply express the number of human resources serving women in disease stage $j$ as ${HW}_{t}^{j2}={HW}_{t}{FHW}_{t}^{j}$, for $j=1,\cdots,L_{w}+1.$

Next state for HIV-infected population

The interim state for HIV-infected men and the distribution of human resources allow us to write the next state at time $t+1$ for HIV-infected men as follows. For $j=1,\cdots,L_{m}+1$,

$M_{t+1}^{j1}=\left( {M^{'}}_{t+1}^{j1}+{M^{'}}_{t+1}^{j2} \right)-\text{min}\left( \varphi{HM}_{t+1}^{j2},{M^{'}}_{t+1}^{j1}+{M^{'}}_{t+1}^{j2} \right)$,

$M_{t+1}^{j2}=\text{min}\left( \varphi{HM}_{t+1}^{j2},{M^{'}}_{t+1}^{j1}+{M^{'}}_{t+1}^{j2} \right)$,

$M_{t+1}^{j3}=\left( {M^{'}}_{t+1}^{j3}+{M^{'}}_{t+1}^{j4} \right)-\text{min}\left( \varphi{HM}_{t+1}^{j4},{M^{'}}_{t+1}^{j3}+{M^{'}}_{t+1}^{j4} \right)$,

$M_{t+1}^{j4}=\text{min}\left( \varphi{HM}_{t+1}^{j4},{M^{'}}_{t+1}^{j3}+{M^{'}}_{t+1}^{j4} \right)$.

Similarly, we can write the next state for HIV-infected women at time $t+1$ in terms of the interim state for HIV-infected women and the distribution of human resources for women. For $j=1,\cdots,L_{w}+1$,

$W_{t+1}^{j1}=\left( {W^{'}}_{t+1}^{j1}+{W^{'}}_{t+1}^{j2} \right)-\text{min}\left( \varphi{HW}_{t+1}^{j2},{W^{'}}_{t+1}^{j1}+{W^{'}}_{t+1}^{j2} \right)$,

$W_{t+1}^{j2}=\text{min}\left( \varphi{HW}_{t+1}^{j2},{W^{'}}_{t+1}^{j1}+{W^{'}}_{t+1}^{j2} \right)$.

Data sources

Tables A–D show the parameter values we use to initialize the model and to control its behavior. The parameters for the baseline model were chosen from the best available evidence for South Africa [18-22]. For the initial distribution of the total population of adult men and women and the inflow into the adult population, we used the empirical data from the national South African population census, which is carried out by the statistics and population census organization of the South African national government, Statistics South Africa [23].

For the number of partners in the last 12 months, we used the empirical data from the South African National HIV, Behaviour and Health Survey, which is carried out in regular intervals by the South African Human Sciences Research Council [20]. For the number of sex acts, we used the South African national data published by the Global Sex Survey [24].

For HIV mortality without ART, we used empirical estimates from South Africa, preceding the roll-out of ART in the South African public-sector health system, which were published by Badri et al. in the *Lancet* in 2006 [19]; for estimates of mortality in the first year of ART, we used empirical estimates published by Braitstein et al. in the *Lancet* in 2006, which included South African data [25].

For time to seroconversion to particular CD4 counts we used several sources, all from sub-Saharan Africa (Table A). For the initial distribution of the HIV-infected population across the different HIV disease stages we use population-based CD4 count data from the Africa Centre for Population Health, a Wellcome Trust-funded research center in South Africa (Table D) [26].

Our estimates of HIV-transmission probabilities are from a meta-analysis by Boily and colleagues [27]. The study reports transmission probabilities by disease stage and separately by sex. We derive sex- and disease-specific transmission probabilities by assuming that equal numbers of women and men were included in the studies whose results were pooled for the summary estimate of transmission probabilities by disease stage, and that the male-female transmission differential does not differ by disease stage. For the reduction in HIV-transmission probability due to ART in all disease stages earlier than CD4 cell count ≥ 200/µl, we use data from the recent HPTN 052 trial [28]. For reduction in HIV-acquisition probability due to circumcision we use 60% per sex act, based on the three large-scale randomized controlled circumcision trials in sub-Saharan Africa [29-32].

Additional modeling details

Acute HIV infection

In this study, we use the results from the 2009 meta-analysis by Boily and colleagues that shows that the transmission probability in the acute phase of the disease is about nine times as high as during the latency period [27] (as listed in Table C). In 2015, new results were published reanalyzing the results from a major study included in the meta-analysis (from Rakai, Uganda), which suggested that previous results substantially overestimated the risk and that the acute phase constitutes less of an impediment to HIV treatment-as-prevention success than previously thought [33]. Our results regarding ART coverage and HIV incidence and mortality reduction are thus likely to be conservative [34]. Additionally, recent modeling results suggest that acute HIV infection does not predict the long-term impact of ART on HIV incidence, even under the assumption of substantially elevated HIV transmission probability in the acute phase [35].

Model replication

With the data tables (Tables A–D) and the mathematical formulation in this SI Appendix, our model is fully specified. Starting from an initial state based on data in Tables A and D and applying the mathematical equations using parameters in Tables B and C, our paper’s results can be replicated for different HRHA scenarios. No other calibrated parameters are used.

Comparison of model results and observed past HIV prevalence trajectory

While our model does not require calibration using abstract parameters, examining the “fit” between model results and observed epidemic trends is interesting. Given an initial state (including an initial HIV prevalence), our model indeed closely reproduces HIV prevalence and population growth trajectories for South Africa from 2003-2012, using estimates for South Africa published by the United Nations organizations. However, comparisons of past prevalence and population growth trends the model would generate if it were run for those past periods merely show that the model is not invalid rather than validate the model. It is pertinent to therefore note that the HIV prevalence projections in our model are also in close agreement with those generated by the other models in recent HIV model comparison studies [12, 14].

Future HIV prevalence and incidence trajectories

We show HIV prevalence trajectories (Figs K and L) and incidence trajectories (Figs M and N) both under the assumption that ART does not have preventive effects and under the assumption that it does lead to reduced HIV transmission. As expected, HIV prevalence and incidence decline more rapidly under the assumption of ART-preventive effects. To understand the epidemiology as presented by the model, we show HIV prevalence and incidence trajectories at different levels of condom coverage. As expected, both prevalence and incidence remain roughly stable at 30% circumcision coverage, when ART does not have preventive effects. HIV prevalence estimates decline substantially with increasing levels of condom coverage [1, 20], both with and without ART prevention effects, as condom coverage reduces HIV incidence levels.

Sensitivity and scenario analyses

The results presented in our paper distill analyses of approximately 200 scenarios designed to explore key uncertainties surrounding the impact of current HRHA numbers on ART coverage and TasP implementation. The analyses suggest that our findings are robust to a wide range of assumptions about ART delivery in practice. The scenarios varied assumptions about HIV transmission probabilities, ART effect on HIV transmission probabilities, ART retention, and ART adherence.

The first assumption we varied concerned the HIV transmission probability per sex act for people receiving ART. The base case assumption is that for a person receiving ART, the transmission probability per sex act is that given by the HPTN 052 study [28] if CD4 cell count ≥ 200 µl and is that given by Boily et al. [27] if the CD4 cell count < 200 µl. Strictly speaking, the HPTN 052 study was conducted only among subjects with CD4 cell counts between 350 µl and 550 µl at ART initiation. However, our analysis indicates the population-level results are not sensitive to assuming the same transmission probability for a person with CD4 cell count between 200–350 µl who is receiving ART.

One reasonable concern about extrapolating from the HPTN 052 results is whether the efficacy of ART observed in that study will be obtained when ART is brought to scale within the population with CD4 cell counts > 350 µl. To see if this concern bears on our conclusions, we examined it under assumptions that the transmission probability for those on ART is two to 10 times higher than observed in the HPTN 052 trial. These variations are high, but we wanted to explore how extreme the violation in assumption would have to be for our results not to hold.

We further systematically varied assumptions to test the robustness of our results related to the retention of people in ART programs and to ART adherence among those who are retained. The base case assumption is that 20% of people who initiate ART are not retained in the program and that 20% of those retained in the program do not adequately adhere to ART [36, 37]. In our model people may return to ART after discontinuing ART at will. When they return to ART, their disease stage is assumed to have progressed depending on how long they have remained off ART. One concern about raising the ART threshold and implementing TasP is whether people in earlier disease stages can be adequately retained and adhere to ART, particularly given the experience with the cascade of care for HIV patients and ART initiation patterns in developing countries [38]. To explore these issues, we increased the ART nonretention and nonadherence rates to 30% and 50% in additional modeling scenarios.

The stability of our results across the wide range of assumptions in these scenarios suggests that increasing ART coverage under current numbers of HRHA is a robust finding, across both variations in the transmission probability assumed for people on ART with CD4 cell count >200 µl and across ART retention and adherence rates (Fig 2 in the main paper). However, different explanations exist for why the rise in ART coverage is insensitive to each parameter. For the transmission probability, the per sex act transmission probability for people receiving ART is so low in earlier disease stages (CD4 cell count > 200 µl) that increasing it 2–10 times its value makes little difference for ART coverage at the population level. The transmission probability of men and women in early disease stages is 0.108% for men and 0.0013% for women (from Table C); thus a 96% reduction gives a very low number for the reduced transmission probabilities (0.004% for men and 0.000052% for women). This transmission probability remains very small even after scaling by a factor of two (0.009% for men and 0.000105% for women) or a factor of 10 (0.043% for men and 0.000524% for women). Our results are thus robust to these changes in transmission probabilities.

However, for ART retention and adherence, the analysis indicates that higher nonretention (and nonadherence) results in larger numbers of deaths and HIV infections. The increase in deaths is much greater than the increase in new infections, so the number of people needing ART shrinks, raising the ART coverage level. However, in this case, the rise in ART coverage stems from two undesirable consequences of ART nonretention and nonadherence, i.e., losses in the potential mortality- and transmission-reducing effects of ART.

Longer time horizons

While prediction uncertainty increases with longer model time horizons, important insights and understanding can nonetheless be gained with long time horizons. In the main paper, we focused the presentation of our results on a comparatively short time horizon, until 2025, compared with many HIV modeling studies. For instance, in one recent model comparison of 12 mathematical models of the HIV epidemic and cost-effectiveness of early ART, the time horizon extended until 2030 [39]. In another recent model comparison of 12 mathematical models of the HIV epidemic and the impact of ART on HIV incidence in South Africa, the time horizon extended until 2050 [40].

We thus here also report our model results to 2040 to examine in particular when universal (100%) ART coverage is reached under different model assumptions and how many HRHA teams are needed to achieve and maintain universal ART coverage over time. We find that the coverage trends observed until 2025 continue until 2040 (Figs C and D). Importantly, in all scenarios with ART-preventive effects universal ART coverage is reached before 2040; in contrast, in the scenario without ART-preventive effects, universal coverage is not reached by 2040, and the coverage difference between this scenario and the scenarios with ART-preventive effects continues to increase (Fig O). Similarly, the trends in the number of HRHA teams needed to maintain universal coverage continue until 2040 (Fig D). Overall, none of our main conclusions is affected by the choice of time horizon. Over a longer time horizons, the main trends and differences are simply more pronounced.

References

1. Shisana O, Rehle T, Simbayi LC, Zuma KJ, Sean, Zungu N, Labadarios D, et al. South African National HIV Prevalence, Incidence and Behaviour Survey, 2012. Cape Town, South Africa: HSRC Press; 2014.

2. WHO, UNAIDS, UNICEF. Global update on HIV treatment 2013: results, impact and opportunities. Geneva, Switzerland: World Health Organization, 2013.

3. PEPFAR. Partnership Framework in Support of South Africa’s National HIV, STI & TB Response (2012/13 – 2016/17). Pretoria, South Africa: Government of South Africa and the Government of the United States of America, 2011.

4. Katz IT, Bassett IV, Wright AA. PEPFAR in Transition — Implications for HIV Care in South Africa. New England Journal of Medicine. 2013;369(15):1385-7. PubMed PMID: 24106930.

5. Bärnighausen T, Bloom DE, Humair S. Economics of antiretroviral treatment vs. circumcision for HIV prevention. Proceedings of the National Academy of Sciences. 2012;109(52):21271-6.

6. Alistar SS, Owens DK, Brandeau ML. Effectiveness and Cost Effectiveness of Expanding Harm Reduction and Antiretroviral Therapy in a Mixed HIV Epidemic: A Modeling Analysis for Ukraine. PLoS Med. 2011;8(3):e1000423.

7. Brown T, Bao L, Raftery AE, Salomon JA, Baggaley RF, Stover J, et al. Modelling HIV epidemics in the antiretroviral era: the UNAIDS Estimation and Projection package 2009. Sexually Transmitted Infections. 2010;86(Suppl 2):ii3-ii10.

8. Lima VD, Johnston K, Hogg RS, Levy AR, Harrigan PR, Anema A, et al. Expanded access to highly active antiretroviral therapy: a potentially powerful strategy to curb the growth of the HIV epidemic. J Infect Dis. 2008;198(1):59-67. Epub 2008/05/24. PubMed PMID: 18498241.

9. Stover J, Bollinger L, Avila C. Estimating the Impact and Cost of the WHO 2010 Recommendations for Antiretroviral Therapy. AIDS Research and Treatment. 2011;2011: 738271.

10. Stover J, Bollinger L, Hecht R, Williams C, Roca E. The impact of an AIDS vaccine in developing countries: a new model and initial results. Health Aff (Millwood). 2007;26(4):1147-58. Epub 2007/07/17. PubMed PMID: 17630459.

11. Stover J, Johnson P, Hallett T, Marston M, Becquet R, Timaeus IM. The Spectrum projection package: improvements in estimating incidence by age and sex, mother-to-child transmission, HIV progression in children and double orphans. Sexually Transmitted Infections. 2010;86(Suppl 2):ii16-ii21.

12. Eaton JW, Johnson LF, Salomon JA, Bärnighausen T, Bendavid E, Bershteyn A, et al. HIV Treatment as Prevention: Systematic Comparison of Mathematical Models of the Potential Impact of Antiretroviral Therapy on HIV Incidence in South Africa. PLoS Med. 2012;9(7):e1001245.

13. Granich RM, Gilks CF, Dye C, De Cock KM, Williams BG. Universal voluntary HIV testing with immediate antiretroviral therapy as a strategy for elimination of HIV transmission: a mathematical model. Lancet. 2009;373(9657):48-57. Epub 2008/11/29. PubMed PMID: 19038438.

14. Eaton JW, Menzies NA, Stover J, Cambiano V, Chindelevitch L, Cori A, et al. Health benefits, costs, and cost-effectiveness of earlier eligibility for adult antiretroviral therapy and expanded treatment coverage: a combined analysis of 12 mathematical models. The Lancet Global Health. 2014;2(1):e23-e34.

15. WHO. HIV Drug Resistance Report 2012. Geneva, Switzerland: World Health Organization, 2012.

16. Gallagher J. HIV evolving 'into milder form'. BBC News. Online ed. London, UK, http://www.bbc.com/news/health-30254697: BBC News; 2014.

17. Payne R, Muenchhoff M, Mann J, Roberts HE, Matthews P, Adland E, et al. Impact of HLA-driven HIV adaptation on virulence in populations of high HIV seroprevalence. Proceedings of the National Academy of Sciences. 2014;111(50):E5393–E400.

18. Adam MA, Johnson LF. Estimation of adult antiretroviral treatment coverage in South Africa. South African Medical Journal. 2009;99(9):661-7.

19. Badri M, Lawn SD, Wood R. Short-term risk of AIDS or death in people infected with HIV-1 before antiretroviral therapy in South Africa: a longitudinal study. Lancet. 2006;368(9543):1254-9. Epub 2006/10/10. PubMed PMID: 17027731.

20. Shisana O, Rehle T, Simbayi LC, Zuma K, Jooste S, Pillay-van-Wyk V, et al. South African national prevalence, incidence, behaviour and communication survey: a turning tide among teenagers? Cape Town, South Africa: HSRC Press; 2009.

21. WHO. Life tables for WHO member states Geneva, Switzerland: World Health Organization; 2009 [12 December 2009]. Available from: http://apps.who.int/whosis/database/life_tables/life_tables.cfm.

22. Statistics South Africa (StatsSA). Mid-year population estimates 2009. Pretoria, South Africa: Statistics South Africa; 2009.

23. Statistics South Africa (StatsSA). Mid-year population estimates 2011. Pretoria, South Africa: Statistics South Africa; 2011.

24. Durex. 2005 Global sex survey. Durex, http://www.data360.org/pdf/20070416064139.Global%20Sex%20Survey.pdf 2005 Contract No.: 12 December 2009.

25. Braitstein P, Brinkhof MW, Dabis F, Schechter M, Boulle A, Miotti P, et al. Mortality of HIV-1-infected patients in the first year of antiretroviral therapy: comparison between low-income and high-income countries. Lancet. 2006;367(9513):817-24. Epub 2006/03/15. PubMed PMID: 16530575.

26. Malaza A, Bärnighausen T, Tanser F, Newell C, Newell M. CD4 distributions and unmet ART need in a general population in rural KwaZulu-Natal. Oral presentation, 5^th^ South African AIDS Conference, 7-10 Jun 2011, Inkosi Albert Luthuli International Convention Centre, Durban, South Africa2011.

27. Boily MC, Baggaley RF, Wang L, Masse B, White RG, Hayes RJ, et al. Heterosexual risk of HIV-1 infection per sexual act: systematic review and meta-analysis of observational studies. Lancet Infect Dis. 2009;9(2):118-29. Epub 2009/01/31. PubMed PMID: 19179227.

28. Cohen MS, Chen YQ, McCauley M, Gamble T, Hosseinipour MC, Kumarasamy N, et al. Prevention of HIV-1 Infection with Early Antiretroviral Therapy. New England Journal of Medicine. 2011;365(6):493-505.

29. Auvert B, Taljaard D, Lagarde E, Sobngwi-Tambekou J, Sitta R, Puren A. Randomized, controlled intervention trial of male circumcision for reduction of HIV infection risk: the ANRS 1265 Trial. PLoS Med. 2005;2(11):e298. Epub 2005/10/20. PubMed PMID: 16231970.

30. Gray RH, Kigozi G, Serwadda D, Makumbi F, Watya S, Nalugoda F, et al. Male circumcision for HIV prevention in men in Rakai, Uganda: a randomised trial. Lancet. 2007;369(9562):657-66. Epub 2007/02/27. PubMed PMID: 17321311.

31. Bailey RC, Moses S, Parker CB, Agot K, Maclean I, Krieger JN, et al. Male circumcision for HIV prevention in young men in Kisumu, Kenya: a randomised controlled trial. Lancet. 2007;369(9562):643-56. Epub 2007/02/27. PubMed PMID: 17321310.

32. Williams BG, Lloyd-Smith JO, Gouws E, Hankins C, Getz WM, Hargrove J, et al. The Potential Impact of Male Circumcision on HIV in Sub-Saharan Africa. PLoS Med. 2006;3(7):e262.

33. Abu-Raddad L. Role of acute HIV infection in driving HIV transmission: implications for HIV treatment as prevention. PLoS Medicine. 2015;12(3):e1001803.

34. Bellan SE, Dushoff J, Galvani AP, Meyers LA. Reassessment of HIV-1 acute phase infectivity: accounting for heterogeneity and study design with simulated cohorts. PLoS Med. 2015;12(3):e1001801. Epub 2015/03/18. PubMed PMID: 25781323; PubMed Central PMCID: PMC4363602.

35. Eaton JW, Hallett TB. Why the proportion of transmission during early-stage HIV infection does not predict the long-term impact of treatment on HIV incidence. Proceedings of the National Academy of Sciences of the United States of America. 2014;111(45):16202-7. Epub 2014/10/15. PubMed PMID: 25313068; PubMed Central PMCID: PMC4234601.

36. Kalichman SC, Cherry C, Amaral CM, Swetzes C, Eaton L, Macy R, et al. Adherence to Antiretroviral Therapy and HIV Transmission Risks: Implications for Test-and-Treat Approaches to HIV Prevention AIDS Patient Care and STDs. 2010;24 (5):271-7.

37. Mutevedzi P, Lessells R, Heller T, Bärnighausen T, Cooke G, Newell ML. Scale-up of a decentralised HIV treatment programme in rural KwaZulu-Natal, South Africa: does rapid expansion affect patient outcomes? Bulletin of the WHO. 2010;88(8):593-600.

38. Gardner EM, McLees MP, Steiner JF, del Rio C, Burman WJ. The Spectrum of Engagement in HIV Care and its Relevance to Test-and-Treat Strategies for Prevention of HIV Infection. Clinical Infectious Diseases. 2011;52(6):793-800.

39. Eaton JW, Menzies NA, Stover J, Cambiano V, Chindelevitch L, Cori A, et al. Health benefits, costs, and cost-effectiveness of earlier eligibility for adult antiretroviral therapy and expanded treatment coverage: a combined analysis of 12 mathematical models. Lancet Glob Health. 2014;2(1):e23-34. Epub 2014/08/12. PubMed PMID: 25104632.

40. Eaton JW, Johnson LF, Salomon JA, Barnighausen T, Bendavid E, Bershteyn A, et al. HIV treatment as prevention: systematic comparison of mathematical models of the potential impact of antiretroviral therapy on HIV incidence in South Africa. PLoS Med. 2012;9(7):e1001245. Epub 2012/07/18. PubMed PMID: 22802730; PubMed Central PMCID: PMC3393664.

41. WHO, UNAIDS, UNICEF. Towards universal access: scaling up priority HIV/AIDS interventions in the health sector. Geneva, Switzerland: World Health Organization; 2009.

42. Todd J, Glynn JR, Marston M, Lutalo T, Biraro S, Mwita W, et al. Time from HIV seroconversion to death: a collaborative analysis of eight studies in six low and middle-income countries before highly active antiretroviral therapy. AIDS. 2007;21 Suppl 6:S55-63. Epub 2008/01/11. PubMed PMID: 18032940.

43. eART-linc. Duration from seroconversion to eligibility for antiretroviral therapy and from ART eligibility to death in adult HIV-infected patients from low and middle-income countries: collaborative analysis of prospective studies. Sex Transm Infect. 2008;84 Suppl 1:i31-i6. Epub 2008/07/25. PubMed PMID: 18647863.

44. Minga A, Coulibaly A, Anglaret X, Dohoun L, Abo Y, Toni T, et al., editors. Evolution to the need of care in HIV-1 seroconverters adults with CD4+ cell count above > 500/mm³. The ANRS 1220 Primo-CI cohort1997-2008, Abidjan, Côte d'Ivoire 5th IAS Conference on HIV Pathogenesis, Treatment and Prevention, 19-22 July 2009; Cape Town.

45. Njeuhmeli E, Forsythe S, Reed J, Opuni M, Bollinger L, Heard N, et al. Voluntary Medical Male Circumcision: Modeling the Impact and Cost of Expanding Male Circumcision for HIV Prevention in Eastern and Southern Africa. PLoS Med. 2011;8(11):e1001132.

46. Filler SJ, Berruti AA, Menzies N, Berzon R, Ellerbrock TV, Ferris R, et al. Characteristics of HIV Care and Treatment in PEPFAR-Supported Sites. JAIDS Journal of Acquired Immune Deficiency Syndromes. 2011;57(1):e1-e6.

47. Bärnighausen T, Bloom DE, Humair S. A mathematical model for estimating the number of health workers required for universal antiretroviral treatment. National Bureau of Economics Research, Cambridge, MA: NBER Working Paper #15517; 2009.

48. Malaza A, Mossong J, Bärnighausen T, Viljoen J, Newell M-L. Population-Based CD4 Counts in a Rural Area in South Africa with High HIV Prevalence and High Antiretroviral Treatment Coverage. PLoS ONE,. 2013;8(7):e70126.

**Table A: Parameters for the base case.**

| **Parameters** | **Men** | **Women** | **Source** |
| --- | --- | --- | --- |
| **Initial distribution (#)** |  |  |  |
| HIV uninfected | 13,798,660 | 14,692,842 | [23] |
| HIV infected | 2,746,496 | 3,536,491 | [23] |
| Distribution into HIV-stages | See Table D |  |  |
| **Annual inflow (#)** |  |  |  |
| HIV uninfected | 522,442 | 492,531 | [23] |
| HIV infected, early stage (first year) | 13,396 | 35,369 | [23] |
| **Time from seroconversion (years)** |  |  |  |
| To CD4<200/µl | 8 | 9 | [41], [42], [43] |
| To CD4<350/µl | 5 | 5 | [44] |
| **Total number of sex acts per year** | 109 | 109 | [24] |
| **Number of partners in last 12 months** |  |  |  |
| Proportion having 1 partner | 0.917 | 0.917 | [20] |
| Proportion having 2 partners | 0.049 | 0.049 | [20] |
| Proportion having 3 partners | 0.034 | 0.034 | [20] |
| **Annual probability of death** | See Table B |  |  |
| **Transmission probability per sex act** | See Table C |  |  |
| **Circumcision coverage** | 45% |  | [45] |
| **ART nonretention and nonadherence** |  |  |  |
| Nonretention on ART | 20% | 20% | [36, 37] |
| Nonadherence to ART | 20% | 20% | [36, 37] |
| **Human resources (HRHA)** |  |  |  |
| Initial number of HRHA teams | 2313 (for the entire HIV-infected population) | |  |
| HRHA team to patient ratio | 1.29 teams per 1000 patients | | [46] |

Table B: Mortality rate (per person per year) in different HIV stages for people receiving and not receiving ART [19, 21, 22, 25].

| **Infection** | **Mortality for men** | | **Mortality for women** | |
| --- | --- | --- | --- | --- |
| **year** | **No ART** | **ART** | **No ART** | **ART** |
| 0 | 0.013 | 0.013 | 0.009 | 0.009 |
| 1 | 0.049 | 0.027 | 0.049 | 0.027 |
| 2 | 0.049 | 0.027 | 0.049 | 0.027 |
| 3 | 0.049 | 0.027 | 0.049 | 0.027 |
| 4 | 0.049 | 0.027 | 0.049 | 0.027 |
| 5 | 0.049 | 0.027 | 0.049 | 0.027 |
| 6 | 0.077 | 0.027 | 0.077 | 0.027 |
| 7 | 0.077 | 0.027 | 0.077 | 0.027 |
| 8 | 0.237 | 0.027 | 0.237 | 0.027 |
| 9 (9+ for men) | 0.237 | 0.027 | 0.237 | 0.027 |
| 10+ (for women) |  |  | 0.237 | 0.027 |

Table C: Transmission probability per sex act for people not receiving and receiving ART. HPTN 052 is from [28].

| **Infection** | **Men’s transmission probability** | | | | **Women’s transmission probability** | | |
| --- | --- | --- | --- | --- | --- | --- | --- |
| **year** | **No ART** | **ART** | **Source** | **No ART** | | **ART** | **Source** |
| ≤0.33 | 0.00987 | 0.0002273 | HPTN 052 | 0.00329 | | 0.0001031 | HPTN 052 |
| 0.33–1 | 0.00401 | 0.0001213 | HPTN 052 | 0.00134 | | 0.0000463 | HPTN 052 |
| 2 | 0.00108 | 0.0000378 | HPTN 052 | 0.00036 | | 0.0000131 | HPTN 052 |
| 3 | 0.00108 | 0.0000378 | HPTN 052 | 0.00036 | | 0.0000131 | HPTN 052 |
| 4 | 0.00108 | 0.0000378 | HPTN 052 | 0.00036 | | 0.0000131 | HPTN 052 |
| 5 | 0.00108 | 0.0000378 | HPTN 052 | 0.00036 | | 0.0000131 | HPTN 052 |
| 6 | 0.00108 | 0.0000378 | HPTN 052 | 0.00036 | | 0.0000131 | HPTN 052 |
| 7 | 0.00108 | 0.0000378 | HPTN 052 | 0.00036 | | 0.0000131 | HPTN 052 |
| 8 | 0.00108 | 0.0000378 | HPTN 052 | 0.00036 | | 0.0000131 | HPTN 052 |
| 9 (9+ for men) | 0.008295 | 0.0010800 | [27] | 0.00036 | | 0.0000131 | HPTN 052 |
| 10+ (for women) |  |  |  | 0.002765 | | 0.0003600 | [27] |

Table D: Initial distribution of the HIV-infected population across CD4 count–defined HIV disease stages based on Malaza et al. [26]. Top table shows data from [26]. Bottom table shows the data mapped to the initial state in our model.

| **HIV disease stage/**  **CD4 count category** | **Total** | **Men** | **Women** |
| --- | --- | --- | --- |
| <200 | 34% | 35% | 34% |
| (200, 350) | 21% | 24% | 20% |
| (350,500) | 17% | 19% | 16% |
| ≥500 | 22% | 17% | 23% |

| **Infection** | **Initial distribution of HIV-infected** | |
| --- | --- | --- |
| **year** | **Men** | **Women** |
| 1 | 10.89% | 10.83% |
| 2 | 10.40% | 10.31% |
| 3 | 9.97% | 9.85% |
| 4 | 9.63% | 9.46% |
| 5 | 9.31% | 9.11% |
| 6 | 9.00% | 8.82% |
| 7 | 8.41% | 8.28% |
| 8 | 7.82% | 7.76% |
| 9 (9+ for men) | 24.57% | 5.98% |
| 10+ (for women) |  | 19.60% |
| Total | 100.0% | 100.0% |

Fig A: Previous result from [47] showing HRHA needed to achieve and indefinitely maintain a desired level of ART coverage.

Fig B: Empirical evidence for how the provision of ART can change CD4 cell count distribution in an HIV-infected population. Proportions of population in different CD4 cell count categories shown for three populations: for ART-naïve population (blue bars), for ART-receiving population (line) and overall HIV-infected population both receiving and not receiving ART (green bars). Of interest is the difference between the blue and green bars. Based on data from a population-based survey conducted in rural South Africa [48]. All numbers show percentage of respective populations in the different CD4 count categories; across CD4 count categories the percentages for each population sum up to 100%. For those receiving ART, CD4 cell count category is at initiation of ART.

Fig C: Trajectory of ART coverage in South Africa if HRHA were to stay constant at the initial level. Coverage shown for two cases: if ART has prevention effects and if ART has no prevention effects.

Fig D: “Surge capacity”: The number of HRHA needed after reaching universal (100%) ART coverage. The figure shows the drawdown in HRHA that is possible while maintaining universal ART coverage for two cases: if ART has prevention effects and if ART has no prevention effects.

Fig E: New infections in the presence of high levels of HRHA outmigration.

Fig F: Deaths in the presence of high levels of HRHA outmigration.

Fig G: Effect on new infections of shifting resources from ART to TasP with 10% ART resistance (in the absence of second-line ART).

Fig H: Effect on new infections of shifting resources from ART to TasP with 30% ART resistance (in the absence of second-line ART).

Fig I: Effect on deaths of shifting resources from ART to TasP with 10% ART resistance (in the absence of second-line ART).

Fig J: Effect on deaths of shifting resources from ART to TasP with 30% ART resistance (in the absence of second-line ART).

Fig K: HIV prevalence trajectories without ART prevention effects, at different levels of condom coverage (cc).

Fig L: HIV prevalence trajectories with ART prevention effects, at different levels of condom coverage (cc).

Fig M: HIV incidence trajectories without ART prevention effects, at different levels of condom coverage (cc).

Fig N: HIV incidence with ART prevention effects, at different levels of condom coverage (cc).

Fig O: Trajectory of ART coverage in South Africa if human resources for treating HIV/AIDS (HRHA) were to stay constant at the initial level (Fig 2 in the main paper over a longer horizon). Coverage shown for the following cases: assuming ART does not have prevention effects; assuming ART prevention effects are as observed in the HPTN 052 trial; ART prevention effects that are 1/2 or 1/10th of that observed in the HPTN 052 trial; and assuming ART nonretention rate is 30% or 50%. The lines for the following three cases are very close and hence all lines are not visible: with ART prevention effect, ART effect on HIV transmission probability 1/2 of that observed in HPTN 052, and ART effect on HIV transmission probability 1/2 of that observed in HPTN 052.

**Fig P: Trajectory of ART coverage in South Africa if human resources for treating HIV/AIDS (HRHA) were to stay constant at the initial level. Shown for alternative HRHA/Patient ratios.**

**Fig Q: “Surge capacity”: The number of HRHA needed after reaching universal (100%) ART coverage.** **Shown for alternative HRHA/Patient ratios.**

**Fig R: “Surge capacity”: The number of HRHA needed after reaching universal (100%) ART coverage.** **Shown for alternative scenarios where HRHA inflows are less than those assumed in base case. Base case annual HRHA inflow is assumed to be 520.**
